# Phylogeographic analysis of severe fever with thrombocytopenia syndrome virus from Zhoushan Islands, China: implication for transmission across the ocean

**DOI:** 10.1038/srep19563

**Published:** 2016-01-25

**Authors:** Yongfeng Fu, Shibo Li, Zhao Zhang, Suqin Man, Xueping Li, Wenhong Zhang, Chiyu Zhang, Xunjia Cheng

**Affiliations:** 1 Institute of Biomedical Sciences, Department of Medical Microbiology and Parasitology, School of Basic Medical Sciences, Fudan University, Shanghai, China; 2 Zhoushan Hospital affiliated to Wenzhou Medical University, Zhejiang, China; 3 Institut Pasteur of Shanghai, Chinese Academy of Sciences, Shanghai, China; 4 School of Life Sciences, Fudan University, Shanghai, China

## Abstract

From June 2011 to August 2014, 21 cases of infection by severe fever with thrombocytopenia syndrome bunyavirus (SFTSV) were confirmed in Zhoushan Islands in the Eastern coast of China. To identify the source of SFTSV in Zhoushan Islands, the whole SFTSV genomes were amplified and sequenced from 17 of 21 patients. The L, M, and S genomic segments of these SFTSV strains were phylogenetically analyzed together with those of 188 SFTSV strains available from GenBank. Phylogenetic analysis demonstrated SFTSV could be classified into six genotypes. The genotypes F, A, and D were dominant in mainland China. Additionally, seven types of SFTSV genetic reassortants (abbreviated as AFA, CCD, DDF, DFD, DFF, FAF, and FFA for the L, M and S segments) were identified from 10 strains in mainland China. Genotype B was dominant in Zhoushan Islands, Japan and South Korea, but not found in mainland China. Phylogeographic analysis also revealed South Korea possible be the origin area for genotype B and transmitted into Japan and Zhoushan islands in the later part of 20^th^ century. Therefore, we propose that genotype B isolates were probable transmitted from South Korea to Japan and Zhoushan Islands.

Severe fever with thrombocytopenia syndrome (SFTS) is a new infectious disease that emerged in May 2007 in rural areas of Huaiyangshan Mountain, which links Hubei and Henan Provinces in Central China^1^,^2^,^3^. SFTS is characterized by fever, thrombocytopenia, gastrointestinal symptoms, and leukocytopenia and has a mortality rate of 12.3–20%^1^,^4^,^5^,^6^,^7^,^8^. The causative agent for SFTS was identified as a novel bunyavirus, which was named SFTS bunyavirus (SFTSV) or Huaiyangshan (HYS) virus^1^,^9^. SFTSV was also isolated from ticks (*Haemaphysalis longicornis*) that were collected from domestic animals in the regions where the patients lived^3^,^9^. Furthermore, SFTSV fragments were amplified from several samples from domestic animals (e.g., sheep, cows, and dogs)^9^, suggesting that ticks could serve as a key vector for SFTSV transmission^3^,^9^.

SFTSV belongs to the *Phlebovirus* genus in the *Bunyaviridae* family^1^,^2^,^3^. Its genome contains three separate RNA segments: S (small), M (middle), and L (large). So far, SFTSV has been isolated only from South Korea, Japan and China. It is prevalent in seven central-eastern provinces of China: Henan, Hubei, Anhui, Jiangsu, Zhejiang, Shandong, and Liaoning^1^,^3^,^4^,^9^,^10^. Zhoushan Islands are located in the Eastern coast of China and geographically isolated from other regions of SFTSV prevalence by the sea. In this study, we sequenced SFTSV isolates from Zhoushan Islands and performed detailed phylogeographical analysis of all SFTSV isolates available from this study and GenBank. This led us to propose that the dominant SFTSV genotype B in Zhoushan Islands was transmitted from South Korea rather than from other parts of China.

## Results

### Demographic and Clinical Characteristics of SFTS patients from the Zhoushan Islands

**Figure 1:**
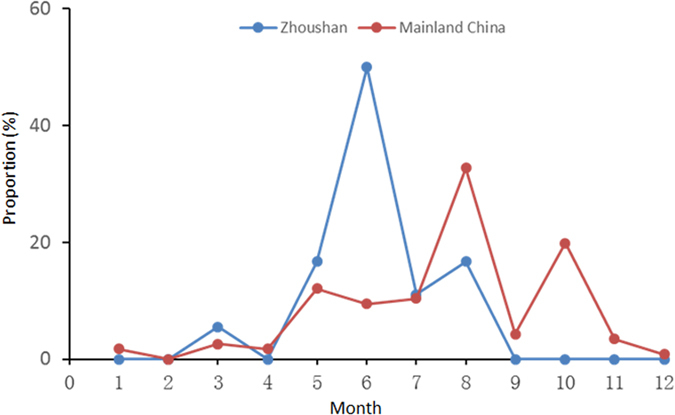
Comparison of monthly distributions of reported SFTS cases between Zhoushan and Mainland China.

During June 2011–August 2014, 21 patients who displayed major clinical symptoms of acute onset of fever, thrombocytopenia, diarrhea, fatigue, and/or cough for 3 to 11 days were diagnosed as SFTS (Table 1). Infection with SFTSV was confirmed by RT-PCR. Symptoms mainly appeared from May–August, and peaked in June (10/21, 47.6%) (Fig.1). All patients, including 18 farmers, 2 fishermen, and 1 gatekeeper, were local residents of the Zhoushan Islands. Their median age was 67.3 (46–86), with 12 males and 9 females. No patient reported tick bites over the previous six months. Laboratory tests indicated that the most common characteristics were thrombocytopenia (100%) and leukocytopenia (95.2%), with elevated levels of serum alanine aminotransferase (100%), aspartate aminotransferase (100%), creatine kinase (100%), and lactate dehydrogenase (100%) (Table 1). The main clinical phenotypes, including fever, diarrhea, leucopenia, thrombocytopenia, were very consistent between patients in Zhoushan and other regions/country (Supplementary Table S1). Fewer patients in Zhoushan felt weakness and abdominal distension than in other regions/country (Supplementary Table S1).

### Classification of SFTSV into six genotypes and identification of reassortants

The whole SFTSV genome was successfully amplified and sequenced from 17 of the 21 patients from Zhoushan. SFTSV genomic segments (L, M, and S) obtained in this study were all deposited in GenBank under the following accession numbers: KR017827–KR017845, KR017846–KR017864, and KR017808–KR017826 (Supplementary Table S2).

**Figure 2:**
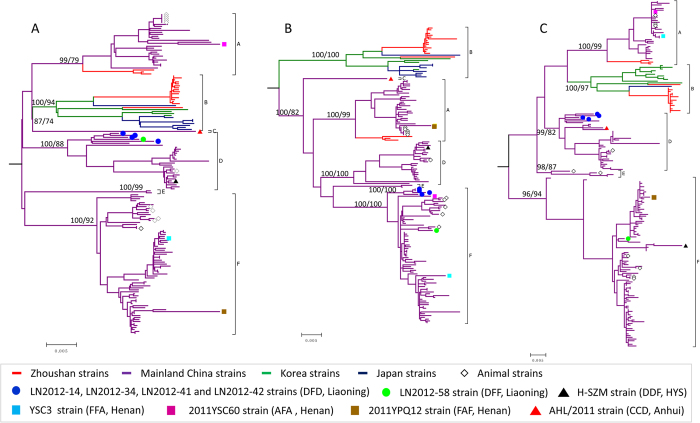
Maximum likelihood (ML) phylogenetic trees of SFTSV L(A), M(B), and S(C) genomic segments. Bootstrap values for ML and NJ tree are shown at corresponding nodes. The red, purple, blue, and green branches represent the strains from Zhoushan, mainland China, Japan, Korea, respectively. The pen diamonds indicate the strains from animals, including ticks, sheep, cows, and dogs. Ten SFTSV reassortants are highlighted by the colored circles, squares, and triangles.

To investigate the genetic relationship between SFTSV strains from the Zhoushan Islands with those from other parts of China and other countries, ML, NJ and MP trees were constructed based on three SFTSV genomic segments of 17 SFTSV strains reported in this study and 188 strains reported previously. Among these SFTSV strains, 159 of which had all the three genomic segment sequenced (Fig. 2). The topological structures derived from ML, NJ, and MP trees of each genomic segment, as well as the tree-topological structures derived from the three genomic segments (L, M, and S), were similar to each other (Fig. 2 and Supplementary Fig. S1). All SFTSV strains were divided into several major clades with high bootstrap supports of ≥85 in the phylogenetic trees, and most strains clustered within the corresponding clades in separate trees of genomic segments L, M, and S (Fig. 2). Based on the phylogeny (Fig. 2) and mean genetic distances of different clades (See Supplementary Table S3 online), six SFTSV genotypes (A–F) were classified. The mean genetic distances within genotypes were 0.001–0.026, and the distances among different genotypes were 0.035–0.062 (See Supplementary Table S3 online). The S genomic segment appeared to be more divergent than the L and M genomic segments.

According to the classification, majority (L: 80%, 132/163; M: 81%, 135/166, and S: 84%, 169/201) of SFTSV strains belong to genotype A, D, or F. All strains from Japan and 4 strains from South Korea and 15 strains from Zhoushan (including 14 obtained in this study and 1 retrieved from GenBank) clustered together, and formed the clade of genotype B (L: 17%, 28/163; M: 16%, 28/166, and S: 13%, 28/201) (Fig. 2). Of the 205 strains, 159 had all the three genomic segments sequenced. Whereas majority (93.7%, 149/159) of strains showed consistent genotype classification among the three genomic segments, ten strains had inconsistent genotype results based on the three genomic segments suggesting reassortment (Fig. 2). Among them, four strains (LN2012–14, LN2012–34, LN2012–41, and LN2012–42) had L and S segments of genotype D, but the M segment of genotype F (abbreviated as DFD for the L, M and S segments), The other six cases were DFF (LN2012-58), FFA (YSC3), AFA (2011YSC60), FAF (2011YPQ12), DDF (H-SZM), and CCD (AHL/2011). Therefore, apart from five SFTSV genotypes (A, B, D, E, and F), there were seven reassortment forms.

### Comparison of SFTSV genotype distribution in China and neighboring countries

**Figure 3:**
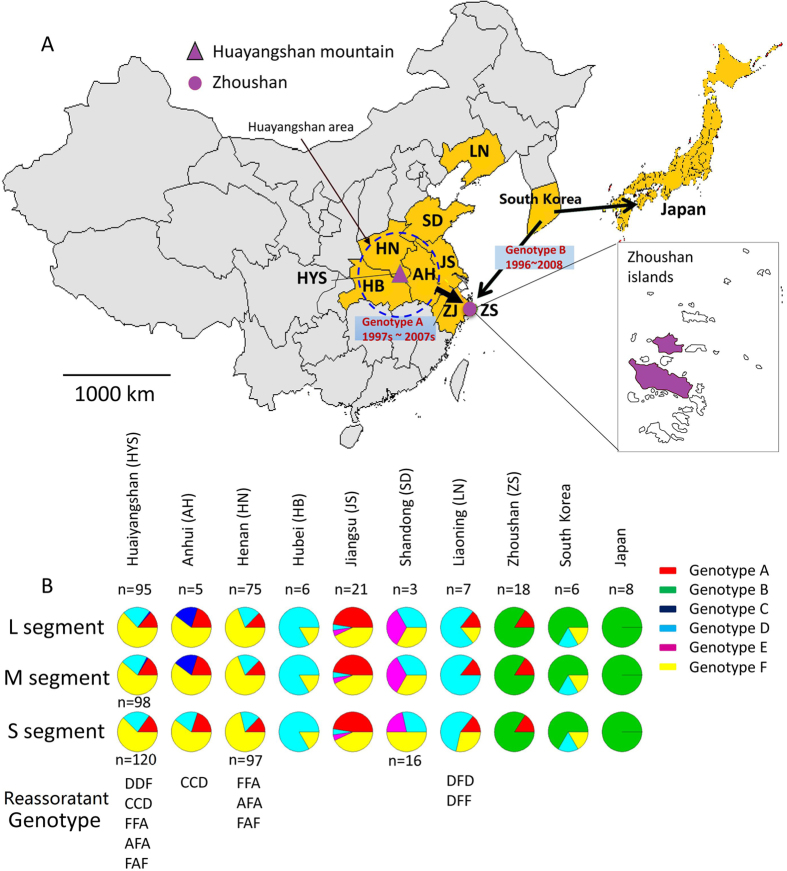
Genotype distribution of SFTSV in China, Korea and Japan. (**A**) The location of Zhoushan Islands, (**B**) Genotype distribution of SFTSV. Arrows indicate the transmission routes of SFTSV genotype B and the transmission routes of SFTSV genotype A from central China to Zhoushan. Two islands where SFTSV was circulating are highlighted in purple. The map was drawn with packages “maps” and “mapdata” in R version 3.1.3(https://cran.r-project.org/web/packages/mapdata/index.html)

The Huaiyangshan Mountain area is located at the junction of Henan, Hubei, and Anhui Provinces and was considered as a single geographic area in this study (Fig. 3A). Genotypes A, D, and F appeared to be the most common SFTSV in mainland China (Fig. 3B). Genotype F was detected in all six affected areas of China. Genotype D was identified in Huaiyangshan, Henan, Hubei, Jiangsu, and Shangdong. Genotypes D and F were also found in South Korea, but not in the Zhoushan Islands or in Japan. Genotype A was found in all targeted provinces/areas in China except Hubei and Shangdong. In Japan, only genotype B was prevalent, and in South Korea, genotypes B, D, and F co-circulated.

Among the pure genotypes, genotype F (43.6%) was the most dominant, followed by genotypes A (20.1%), B (19.5%), and D (15.4%). Genotypes F, A, and D were prevalent in most provinces/areas, and almost all SFTSV strains isolated from animals (e.g., ticks, sheep, cows, and dogs) belonged to these genotypes (A: 45.5%; F: 45.5%; D: 9.0%) (Fig. 3). These findings suggest that the predominant SFTSV genotypes in China had the ability of cross-species transmission between humans and animals (especially ticks). Genotype E was only found in Jiangsu and Shandong, and no pure genotype C strain was found in this study. The SFTSV reassortants were located in Liaoning and Huaiyangshan (including Henan and Anhui).

Genotype B appeared to be only prevalent in islands (Zhoushan and Japan) and/or the Korean peninsula. Apart from genotype B, genotype A was also found in Zhoushan, and genotypes D and F in South Korea.

### Tempospatial dynamics of SFTSV and the origin of the Zhoushan strains

Bayesian analysis was performed to estimate the SFTSV evolutionary rates and timescales. The evolutionary rates for the L, M, and S segments of SFTSV were estimated to be 1.87 (95% HPD, 0.84–3.02), 2.84 (95% HPD, 1.53–4.64), and 5.07 (95% HPD, 2.98–7.09) ×10^−4^ nt substitutions/site/year, respectively (Table 2). Genomic sequences that have exact sample time and geographic area were used to perform the temporal dynamics analyses. Temporal dynamics showed that all SFTSV strains had the time of origin of the most recent common ancestor (tMRCA) at 1868 (95% HPD, 1745–1940), 1867 (95% HPD, 1735–1933), and 1930 (95% HPD, 1880–1963) for the L, M, and S genomic segments, respectively (Table 2, See Supplementary Fig S2 online), suggesting that SFTSV originated during the years 1867–1930. tMRCA of each SFTSV genotype is shown in Table 2. Two SFTSV genotypes (A and B) circulated in Zhoushan. Genotype A of Zhoushan strains cluster together and form a monophyletic lineage with tMRCAs of 1997–2007 (Table 2). Their ancestral geographic states were estimated to be the Huaiyangshan area [posterior probability (PP): 0.99–1, See Supplementary Fig. S2 online], indicating that genotype A was introduced into Zhoushan from central China. For the genotype B strains circulating in Zhoushan, 14 strains (93.3%) formed a monophyletic lineage (Zhoushan linage), and another clustered with a South Korea strain, indicating that genotype B strains were introduced to Zhoushan by two independent events. The inferred ancestral geographic state of the Zhoushan genotype B lineage is South Korea (PP: 0.98–1) and the tMRCA was 1996–2008 (Table 2, See Supplementary Fig. S2 online). Similar to Zhoushan B strains, majority (88.9%) of Japanese genotype B strains formed a monophyletic clade, and another clustered with Zhoushan lineage clade. Their ancestral geographic states were also in South Korea (PP: 0.89–0.94). tMRCA of the Japanese lineage was 1974–1998 (See Supplementary Fig. S2 online).

## Discussion

SFTS was first identified in 2007, and its caustic agent was recognized in 2011 as a novel member of bunyavirus (SFTSV)^1^,^2^,^3^. SFTSV circulated in seven central-eastern provinces of China. Recently it was also found in South Korea and Japan^11^,^12^,^13^, which are separated from China by ocean straits. During June 2011, we reported the first SFTSV case in the Zhoushan Islands^14^. A retrospective review of the local clinic records revealed approximately 15 SFTS-like cases annually in the past decade in the Zhoushan Islands^14^. Therefore, SFTSV may have been epidemic in the Zhoushan Islands for a long period of time.

In this study, we investigated 100 suspected SFTS cases in the Zhoushan Islands from June 2011 to August 2014, and confirmed 21 as SFTSV infection by RT-PCR (Table 1). Most SFTSV infection in the Zhoushan Islands occurred during May–August and peaked in June. In other areas of China, SFTSV infections often occur during May–October, peaking in August (Fig. 1, See Supplementary Table S2 online). Patients in the Zhoushan Islands displayed similar clinical phenotypes and clinical laboratory-parameters similar to those of patients in the mainland areas (Table 1, Supplementary Table S1 online)^1^,^3^,^4^. However, fewer Zhoushan patients had symptoms of weakness and abdominal distension compared to those in other regions of China and Japan^12^,^15^. We were unable to determine whether the clinical symptoms were associated with infection during different SFTSV genotypes since the detail clinical information of each patient reported in previous studies was not available.

Age was an important risk factor for SFTSV infection. The medium age of SFTSV patients in the Zhoushan Islands was 67.3 which similar to that of patients in Japan, and obviously higher than that of patients (52.9–57.2) and fatal cases (62–63) in the mainland areas^2^,^4^,^12^,^16^. All 21 patients recovered after treatment with symptomatic and supportive therapy under the national guideline for SFTS, which may be attributed to the significant improvement in therapy and patient management.

We amplified and sequenced SFTSV genomic sequences circulating in the Zhoushan Islands, and performed phylogenetic analysis with all available genomic sequences from the mainland areas and surrounding countries (South Korea and Japan). Phylogenies of three genomic segments of SFTSV showed six well-supported clades defined as six SFTSV genotypes A–F (Fig. 2). Among 159 SFTSV strains with all the three genomic segments available, 149 belonged to genotypes A, B, D, E and F. Genotypes F, A, B, and D accounted for majority of SFTSV strains. Genotypes D, F, and A co-circulated in wide geographic regions of mainland China, and also accounted for all strains isolated from animals (e.g. ticks and sheep). Remarkably, genotype B circulated only in Japan, the Korean peninsula and Zhoushan Islands of China (Fig. 3B).

RNA viruses are characterized by a high mutation rate and a high potential of recombination, leading to a high genomic heterogeneity of RNA viruses^17^. Reassortment of genomic segments is another important mechanism that increases genetic diversity of segmented viruses (e.g., influenza viruses)^18^. A previous study reported two cases of SFTSV reassorants^18^,^19^. Here we identified 10 SFTSV reassortants that cover 7 reassortment forms (AFA, CCD, DDF, DFD, DFF, FAF, and FFA) (Figs 2 and 3), accounting for 6.3% of SFTSV strains (6.3%, 10/159). Except for DFD that was represented by four reassortant strains, each reassortment form had one representative strain. Majority of these reassortants involved genotypes D, F, and A, which may be explained by high prevalence and co-circulation of the three genotypes in SFTS-affected regions.

Three genomic segments of SFTSV appear to have different evolutionary rates. The S segment underwent more rapid evolution than the L and M genomic segments (Table 2). The time of MRCA (tMRCA) of the SFTSV strains was estimated at 1868, 1867, and 1930 based on L, M, and S genomic segments, respectively. Our estimates on origin time of SFTSV are more recent than a previous report^19^. The most likely reason for the difference was that the restricted molecular clock model was used in the previous study. However, the restricted molecular clock model is not the best fit model to infer the evolution of SFTSV (See Supplementary Table S4 online)^20^,^21^,^22^. The origin time of SFTSV based on L and M segments were very close (1867–1868). Similar to the previous report, the origin time based on the S segment (1930) was obviously more recent than those based on both L and M segments. One possible explanation is that there was no available genotype C sequence in S segment analysis since genotype C may be more ancient than other genotypes, as observed on the temporal dynamic of the L genomic segment (See Supplementary Fig. S2A online).

tMRCA of four predominate genotypes A, B, D, and F were estimated to be 1933–1960, 1901–1924, 1928–1951, and 1944–1971, respectively (Table 2). Genotype B diverged relatively earlier than other genotypes. Geographic origin of all SFTSV genotypes was estimated to most likely be the Huanyangshan area (PP: 0.38–0.82), suggesting that SFTSV spread to other regions of China or surrounding countries from the Huaiyangshan area (Fig. 3A). Two genotypes (A and B) were co-circulating in the Zhoushan Islands. Genotype A strains formed a Zhoushan lineage, having a common geographic origin in the Huaiyangshan area with other genotype A lineages (PP: 0.799–1) (See Supplementary Fig. S2 online). The time for the introduction of genotype A strains into Zhoushan was estimated to be 1997–2007.

Genotype B was only circulating in Zhoushan, South Korea, and Japan. All genotype B strains probably had a geographic origin in South Korea (PP: 0.71–0.82) with tMRCA of 1901–1924 (See Supplementary Fig. S2 online). In the genotype B clade, majority of the Zhoushan and Japanese strains formed their independent lineages and tMRCA of the Zhoushan lineage (1996–2008) was more recent than that of Japanese lineage (1974–1998). Both lineages had a geographic origin of South Korea, implying that the genotype B strain was transmitted from South Korea to the Zhoushan Islands and Japan. In addition, one Japanese strain clustered with the Zhoushan lineage and one Zhoushan strain clustered with one South Korea strain, suggesting at least two independent sea-crossing transmission events of genotype B from South Korea to Zhoushan and from South Korea to Japan. Animals, especially ticks, are the crucial vectors for SFTSV transmission. It is not easy for viruses to spread across geographical barriers, as they do on the continent, because the ocean channels form a natural barrier for most animal reservoirs. International travel increases the possibility of sea-crossing transmission of viruses, and may provide an explanation for the South Korea-to-Japan transmission of SFTSV; however, it is unable to explain how the virus spread to the Zhoushan Islands from South Korea, since almost all SFTSV patients in Zhoushan are local indigenous people and never left the island previously.

Ticks are widely distributed in the world. At least two tick species, *Haemaphysa lislongicornis* and *Rhipicephalus microplu*carry^1^,^9^, transmit SFTSV and other tick-borne pathogens to other animals, including mammals, land birds, and seabirds^23^,^24^,^25^,^26^,^27^. There are many islands in China, South Korea, and Japan, which provide habitats for many migratory seabirds from South Korea to China or other countries, and some ticks with seabirds are dispersed in China, South Korea, and Japan^26^. The Zhoushan Islands attract many seabirds each year. Some seabirds inhabiting the Zhoushan Islands can serve as an indirect vector to spread SFTSV or other tick-borne viruses. We suspect that the SFTSV genotype B likely transmitted from South Korea to Zhoushan and/or Japan with seabird migration across the oceans. To confirm this hypothesis, the samples of ticks of migratory birds should be collected for the detection and sequence analysis of SFTSV in future. According to this hypotheses, SFTSV genotype B should circulate in some coastal regions in China (e.g., Shandong peninsula and Liaoning), which contain natural habitats for migratory seabirds. Therefore, a molecular epidemiological investigation of SFTSV focusing on Chinese coastal regions will not only shed light on SFTSV evolution and transmission, but also provide valuable information for the prevention and control of SFTS in mainland China.

## Methods

### Clinical samples and laboratory testing

Among 100 suspected SFTS cases admitted to Zhoushan People’s Hospital during June 2011–August 2014, 21 were diagnosed as SFTS. In these cases the presence of SFTSV genome was detected by reverse transcription-polymerase chain reaction (RT-PCR) (See Supplementary Methods online). Clinical history and physical examination, routine clinical, biochemical, and hematological laboratory results, and acute phase serum samples were collected from all patients.

This study was conducted according to the Helsinki II Declaration and was approved by the ethics committee at the National Institute of Parasitic Diseases, Chinese Center for Disease Control and Prevention. Written informed consent was obtained from the patients.

### Phylogenetic analysis

All available SFTSV sequences (including those isolated from animals) were directly downloaded from GenBank or blasted in the GenBank database using known L, M, and S sequences of SFTSV as references. In total, 530 sequences from 205 strains (L: 163; M: 166; S: 201) included 17 strains from Zhoushan Island were obtained (See Supplementary Table S2 online). Sequences generated from this study and retrieved from GenBank were divided into L, M, and S datasets for separate phylogenetic analysis. The sequences in each dataset were aligned using the MUSCLE algorithm implemented in MEGA 6.0 and edited manually^28^. Maximum likelihood (ML), Maximum Parsimony (MP) and neighbor-joining (NJ) trees were reconstructed by MEGA 6.0. The best-fitting model for ML analysis was determined using JmodelTest^29^. The ML trees of SFTSV L and M segments were inferred under General Time Reversible model incorporating invariant sites and a gamma distribution (GTR + I + G) and the ML tree of S segment was under Hasegawa-Kishino-Yano (HKY) model. Tree reliability was evaluated by the bootstrap method with 100 replications. For each dataset, mean genetic distances within and between different SFTSV genotypes were calculated using MEGA 6.0^28^.

To estimate SFTSV temporal dynamic, maximum clade credibility (MCC) trees were constructed using a MCMC (Markov Chain Monte Carlo) method implemented in the BEAST v1.8.2 package^20^,^30^. The sequences with known sampling time and geographic location were used in the analysis. The evolutionary rates and the times to MRCA (tMRCA) of various nodes on the MCC tree were also estimated using the BEAST package. A relaxed molecular clock with an uncorrelated lognormal distribution and a constant population size model was used in the Bayesian coalescence analysis. The GTR + Γ 4 + I model of nucleotide substitution was used in the analyses of L and M segments, and the HKY + Γ 4 + I model in the analysis of S segment. Statistical uncertainty in parameter estimates was reflected by the 95% highest posterior density (HPD) values. MCMC analysis was run for 200 million generations for L, M, and S segments with sampling every 10,000 generations to achieve parameter convergence and adequate effective sample sizes (ESS > 200). We summarized the trees using Tree Annotator implemented in the BEAST v1.8.2 package. The initial 25% samples were discarded as burn-in, leaving 75% trees per run to produce consistent tree topologies.

## Additional Information

**How to cite this article**: Fu, Y. *et al.* Phylogeographic analysis of severe fever with thrombocytopenia syndrome virus from Zhoushan Islands, China: implication for transmission across the ocean. *Sci. Rep.* **6**, 19563; doi: 10.1038/srep19563 (2016).

## Supplementary Material

Supplementary Information

## Figures and Tables

**Table 1:** Results on clinical tests in 21 SFTS patients from Zhoushan islands.

Casenumber	Sex	Age(years)	Occupation	Symptoms	Days withsymptoms	Clinical tests						
						WBC(10^9^/L)	PLT(10^9^/L)	HGB(g/L)	AST(U/L)	ALT(U/L)	CK(U/L)	LDH(U/L)
ZS01	F	69	Farmer	Fever with thrombocytopenia	11	4.4	35	89	137	57	573	1336
ZS02	F	61	Farmer	Fever and weakness	5	0.8	20	106	125	100	245	569
ZS03	M	62	Gatekeeper	Fever	6	1	35	110	122	77	737	484
ZS04	M	77	Farmer	Fever, abdominal distension, and feeling restless (10 days)	3	1.7	20	98	556	159	2635	1319
ZS05	M	53	Farmer	Fever and cough	6	1.1	22	126	106	128	437	266
ZS06	M	47	Fishermen	Fever	5	1.9	35	133	715	289	6161	898
ZS07	M	70	Fishermen	Fever and weakness	5	1.4	27	119	91	86	1129	369
ZS08	F	80	Farmer	Fever	6	1.6	42	127	127	63	515	382
ZS09	M	54	Farmer	Pain in back, waist and lower limbs, weakness, and diarrhea	7	1.7	46	118	620	239	2361	660
ZS10	F	66	Farmer	Fever	7	0.6	12	77	437	132	8495	1815
ZS11	M	69	Farmer	Fever and chilly sensations	7	2.6	14	149	379	176	1722	628
ZS12	M	46	Farmer	Fever, cough and diarrhea	6	1.7	21	134	484	182	2346	1478
ZS13	F	59	Farmer	Fever	4	1	91	108	50	60	122	281
ZS14	F	85	Farmer	Fever and diarrhea	7	0.8	33	76	87	56	734	547
ZS15	F	70	Farmer	Fever and diarrhea	4	1.5	34	78	281	108	319	946
ZS16	M	81	Farmer	Fever	3	1.6	34	96	216	118	653	946
ZS17	F	86	Farmer	Fever and diarrhea (5 days)	7	1.67	38	126	592	236	3252	2178
ZS18	F	77	Farmer	Fever	3	0.6	35	88	145	61	273	1187
ZS19	M	55	Farmer	Fever and weakness	5	1.3	19	89	400	125	3554	1594
ZS20	M	69	Farmer	Fever, weakness and diarrhea	5	1.8	20	135	860	232	3523	2415
ZS21	M	71	Farmer	Fever	9	1.1	8	60	1201	516	1514	997

Abbreviations: WBC, white blood cell; PLT, platelet; HGB, hemoglobin; AST, aspartate aminotransferase; ALT, alanine aminotransferase; CK, creatine kinase; LDH, lactate dehydrogenase.

**Table 2:** Bayesian estimates of evolutionary parameters based on L, M, and S genomic fragments of SFTSV.

	L segment	M segment	S segment
Sequence number	126	129	166
Sampling time interval	2007–2014	2007–2014	2007–2014
Coefficient of variation	0.26	0.36	0.80
Evolutionary rate			
(10*-4 nucleotide substitutions/site/year)	1.87 (0.84–3.02)	2.84 (1.53–4.64)	5.07 (2.98–7.09)
tMRCA of SFTSV	1868.5[1745.1–1940]	1867.6[1735.7–1933.2]	1930[1880.3–1963.5]
tMRCA of SFTSV genotype A	1933.5[1865.6–1971.8]	1960.3[1917.5–1984.4]	1962.2[1934.9–1991.5]
tMRCA of SFTSV genotype B	1901.4[1807.7–1957.5]	1924[1848.5–1965.6]	1971.4[1924.6–1982.9]
tMRCA of SFTSV genotype D	1928.4[1857–1966.8]	1951.5[1895.2–1978.7]	1982.1[1959.4–1995.5]
tMRCA of SFTSV genotype E	2005.4[1999.6–2008.6]	2004.3[1996.4–2008.1]	1987.7[1966.6–2002.7]
tMRCA of SFTSV genotype F	1944.5[1888–1978.2]	1971.3[1935–1991.7]	1978.5[1961–1997.3]
Zhoushan lineage of genotype A	1997[1981.4–2005.8]	1997[1979.3–2006.2]	2007.3[1999–2011.1]
Zhoushan lineage of genotype B	2004.1[1996.1–2008.6]	1996.8[1979.9–2005.9]	2008.2[2004.5–2010.4]
Japan lineage of genotype B	1974[1938.8–1993.3]	1990.3[1946–1998.2]	1998.45[1986.9–2005.6]

The 95% highest posterior density credible regions are given in parentheses.
