# Supplementary material for: Phylogeographic analysis of severe fever with thrombocytopenia syndrome virus from Zhoushan Islands, China: implication for transmission across the ocean
Source: Sci Rep. 2016 Jan 25;6:19563. doi: 10.1038/srep19563 (PMC4726339; doi:10.1038/srep19563)
Supplement: Supplementary Information [file srep19563-s1.pdf]

Phylogeographic analysis of severe fever with thrombocytopenia syndrome virus from Zhoushan Islands, China: implication for transmission across the ocean

Yongfeng Fu[1], Shibo Li[1], Zhao Zhang[1], Suqin Man, Xueping Li, Wenhong Zhang, Chiyu Zhang[2] and Xunjia Cheng[2]

1 These authors contributed equally to this work.

2 Corresponding authors

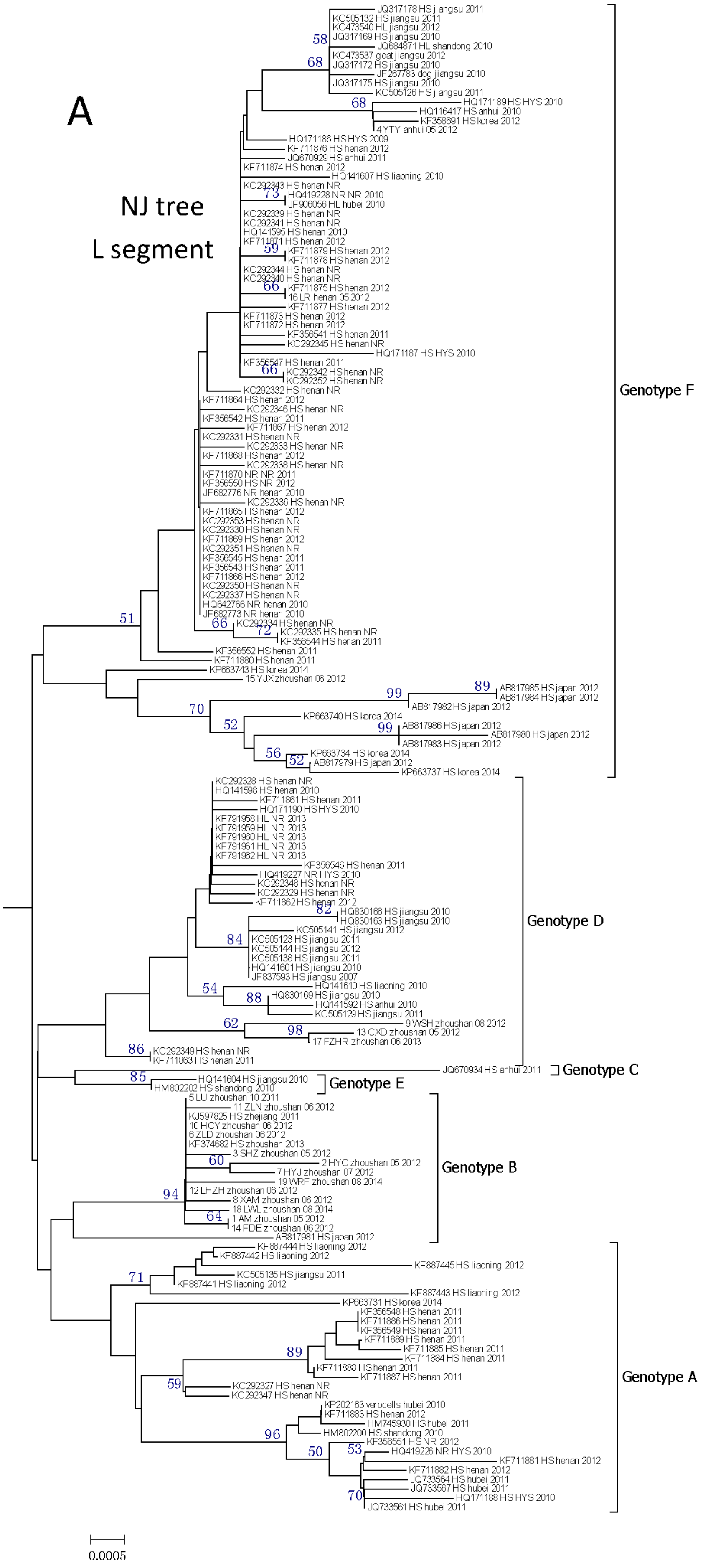
A
NJ tree
L segment
JQ317178 HS jiangsu 2011
KC505132 HS jiangsu 2011
KC473540 HL jiangsu 2012
JQ317169 HS jiangsu 2010
JQ684871 HL shandong 2010
KC473537 goat jiangsu 2012
JQ317172 HS jiangsu 2010
JF267783 dog jiangsu 2010
JQ317175 HS jiangsu 2010
KC505126 HS jiangsu 2011
HQ171189 HS HYS 2010
HQ116417 HS anhui 2010
KF358691 HS korea 2012
4 YTY anhui 05 2012
HQ171186 HS HYS 2009
KF711876 HS henan 2012
JQ670929 HS anhui 2011
KF711874 HS henan 2012
HQ141607 HS liaoning 2010
KC292343 HS henan NR
HQ419228 NR NR 2010
JF906056 HL hubei 2010
KC292339 HS henan NR
KC292341 HS henan NR
HQ141595 HS henan 2010
KF711871 HS henan 2012
KF711879 HS henan 2012
KF711878 HS henan 2012
KC292344 HS henan NR
KC292340 HS henan NR
KF711875 HS henan 2012
16 LR henan 05 2012
KF711877 HS henan 2012
KF711873 HS henan 2012
KF711872 HS henan 2012
KF356541 HS henan 2011
KC292345 HS henan NR
HQ171187 HS HYS 2010
KF356547 HS henan 2011
KC292342 HS henan NR
KC292352 HS henan NR
KC292332 HS henan NR
KF711864 HS henan 2012
KC292346 HS henan NR
KF356542 HS henan 2011
KF711867 HS henan 2012
KC292331 HS henan NR
KC292333 HS henan NR
KF711868 HS henan 2012
KC292338 HS henan NR
KF711870 NR NR 2011
KF356550 HS NR 2012
JF682776 NR henan 2010
KC292336 HS henan NR
KF711865 HS henan 2012
KC292353 HS henan NR
KC292330 HS henan NR
KF711869 HS henan 2012
KC292351 HS henan NR
KF356545 HS henan 2011
KF356543 HS henan 2011
KF711866 HS henan 2012
KC292350 HS henan NR
KC292337 HS henan NR
HQ642766 NR henan 2010
JF682773 NR henan 2010
KC292334 HS henan NR
KC292335 HS henan NR
KF356544 HS henan 2011
KF356552 HS henan 2011
KF711880 HS henan 2011
KP663743 HS korea 2014
15 YJX zhoushan 06 2012
AB817985 HS japan 2012
AB817984 HS japan 2012
AB817982 HS japan 2012
KP663740 HS korea 2014
AB817986 HS japan 2012
AB817980 HS japan 2012
AB817983 HS japan 2012
KP663734 HS korea 2014
AB817979 HS japan 2012
KP663737 HS korea 2014
Genotype F
KC292328 HS henan NR
HQ141598 HS henan 2010
KF711861 HS henan 2011
HQ171190 HS HYS 2010
KF791958 HL NR 2013
KF791959 HL NR 2013
KF791960 HL NR 2013
KF791961 HL NR 2013
KF791962 HL NR 2013
KF356546 HS henan 2011
HQ419227 NR HYS 2010
KC292348 HS henan NR
KC292329 HS henan NR
KF711862 HS henan 2012
HQ830166 HS jiangsu 2010
HQ830163 HS jiangsu 2010
KC505141 HS jiangsu 2012
KC505123 HS jiangsu 2011
KC505144 HS jiangsu 2012
KC505138 HS jiangsu 2011
HQ141601 HS jiangsu 2010
JF837593 HS jiangsu 2007
HQ141610 HS liaoning 2010
HQ830169 HS jiangsu 2010
HQ141592 HS anhui 2010
KC505129 HS jiangsu 2011
9 WSH zhoushan 08 2012
13 CXD zhoushan 05 2012
17 FZHR zhoushan 06 2013
KC292349 HS henan NR
KF711863 HS henan 2011
Genotype D
JQ670934 HS anhui 2011
Genotype C
HQ141604 HS jiangsu 2010
HM802202 HS shandong 2010
Genotype E
5 LU zhoushan 10 2011
11 ZLN zhoushan 06 2012
KJ597825 HS zhejiang 2011
10 HCY zhoushan 06 2012
6 ZLD zhoushan 06 2012
KF374682 HS zhoushan 2013
3 SHZ zhoushan 05 2012
2 HYC zhoushan 05 2012
7 HYJ zhoushan 07 2012
19 WRF zhoushan 08 2014
12 LHZH zhoushan 06 2012
8 XAM zhoushan 06 2012
18 LWL zhoushan 08 2014
1 AM zhoushan 05 2012
14 FDE zhoushan 06 2012
AB817981 HS japan 2012
Genotype B
KF887444 HS liaoning 2012
KF887442 HS liaoning 2012
KF887445 HS liaoning 2012
KC505135 HS jiangsu 2011
KF887441 HS liaoning 2012
KF887443 HS liaoning 2012
KP663731 HS korea 2014
KF356548 HS henan 2011
KF711886 HS henan 2011
KF356549 HS henan 2011
KF711889 HS henan 2011
KF711885 HS henan 2011
KF711884 HS henan 2011
KF711888 HS henan 2011
KF711887 HS henan 2011
KC292327 HS henan NR
KC292347 HS henan NR
KP202163 verocells hubei 2010
KF711883 HS henan 2012
HM745930 HS hubei 2011
HM802200 HS shandong 2010
KF356551 HS NR 2012
HQ419226 NR HYS 2010
KF711881 HS henan 2012
KF711882 HS henan 2012
JQ733564 HS hubei 2011
JQ733567 HS hubei 2011
HQ171188 HS HYS 2010
JQ733561 HS hubei 2011
Genotype A
0.0005

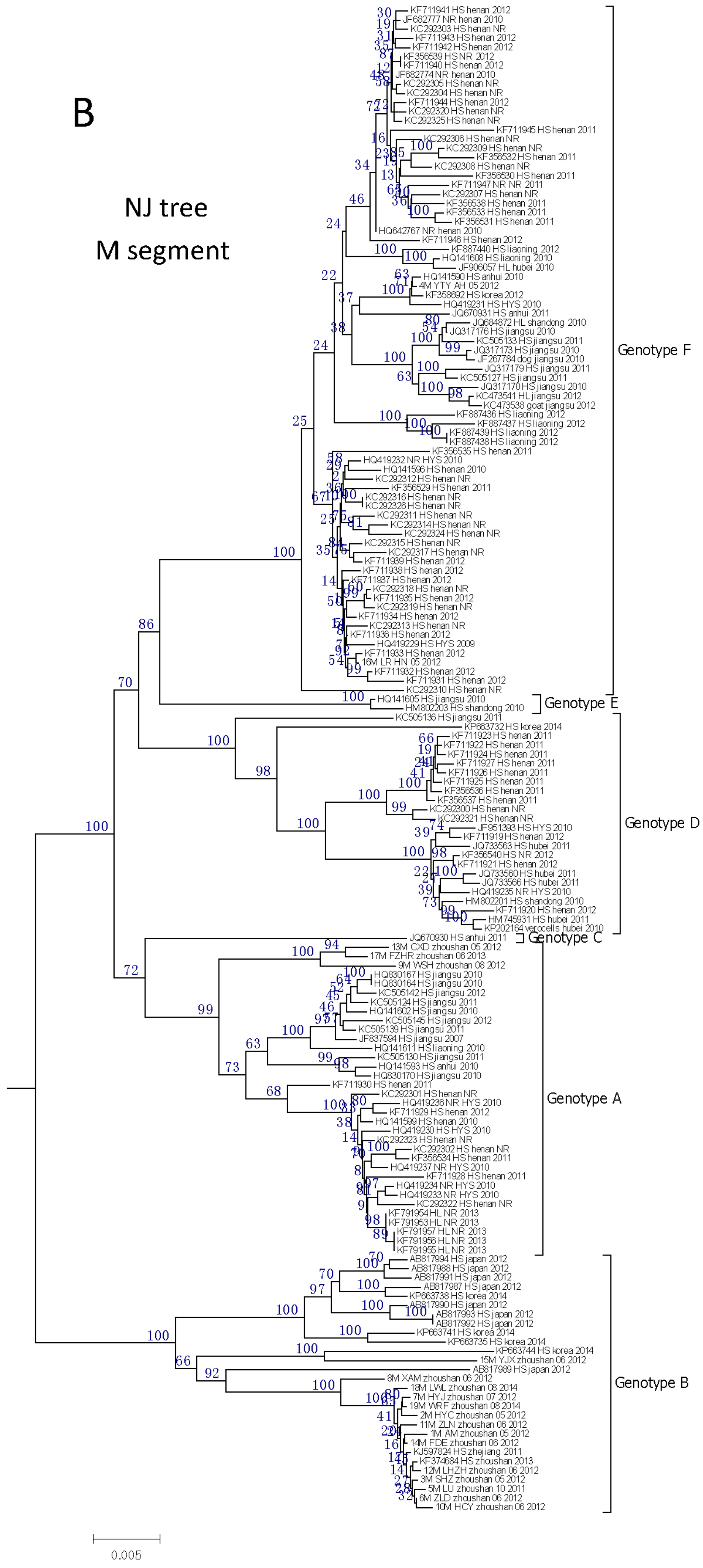
B
NJ tree
M segment
Genotype F
Genotype E
Genotype D
Genotype C
Genotype A
Genotype B
0.005

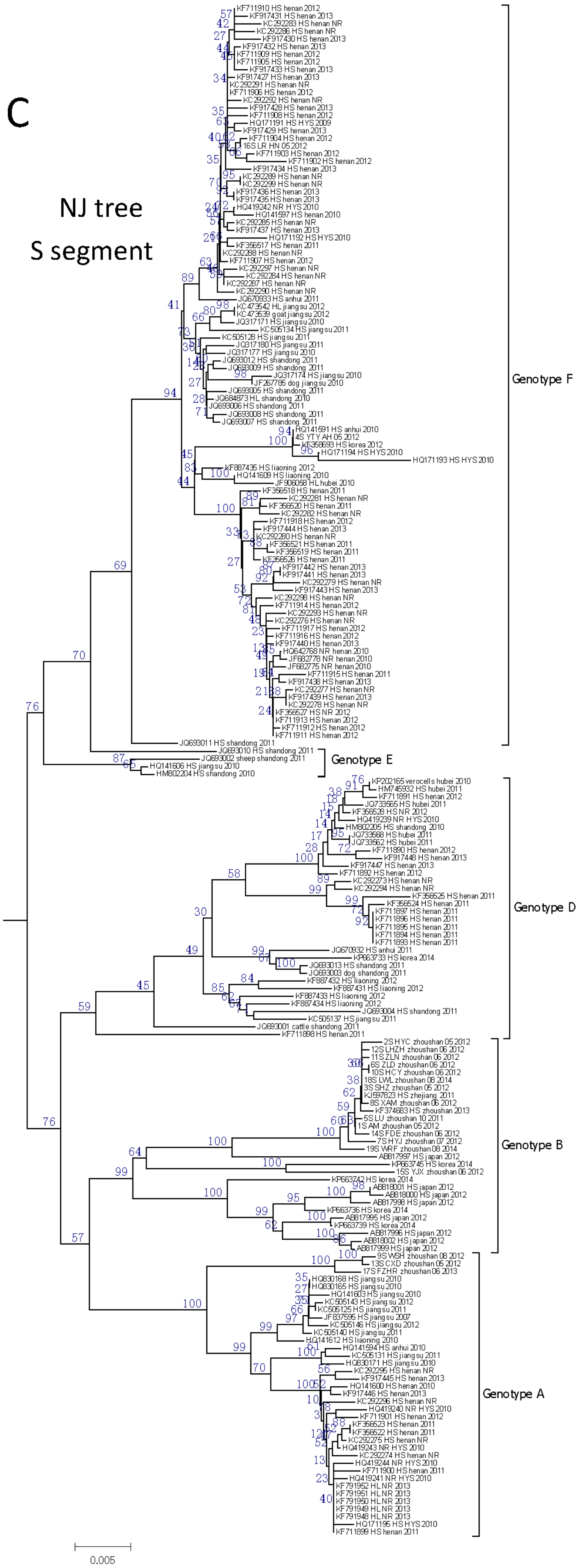
C
NJ tree
S segment
Genotype F
Genotype E
Genotype D
Genotype B
Genotype A
0.005

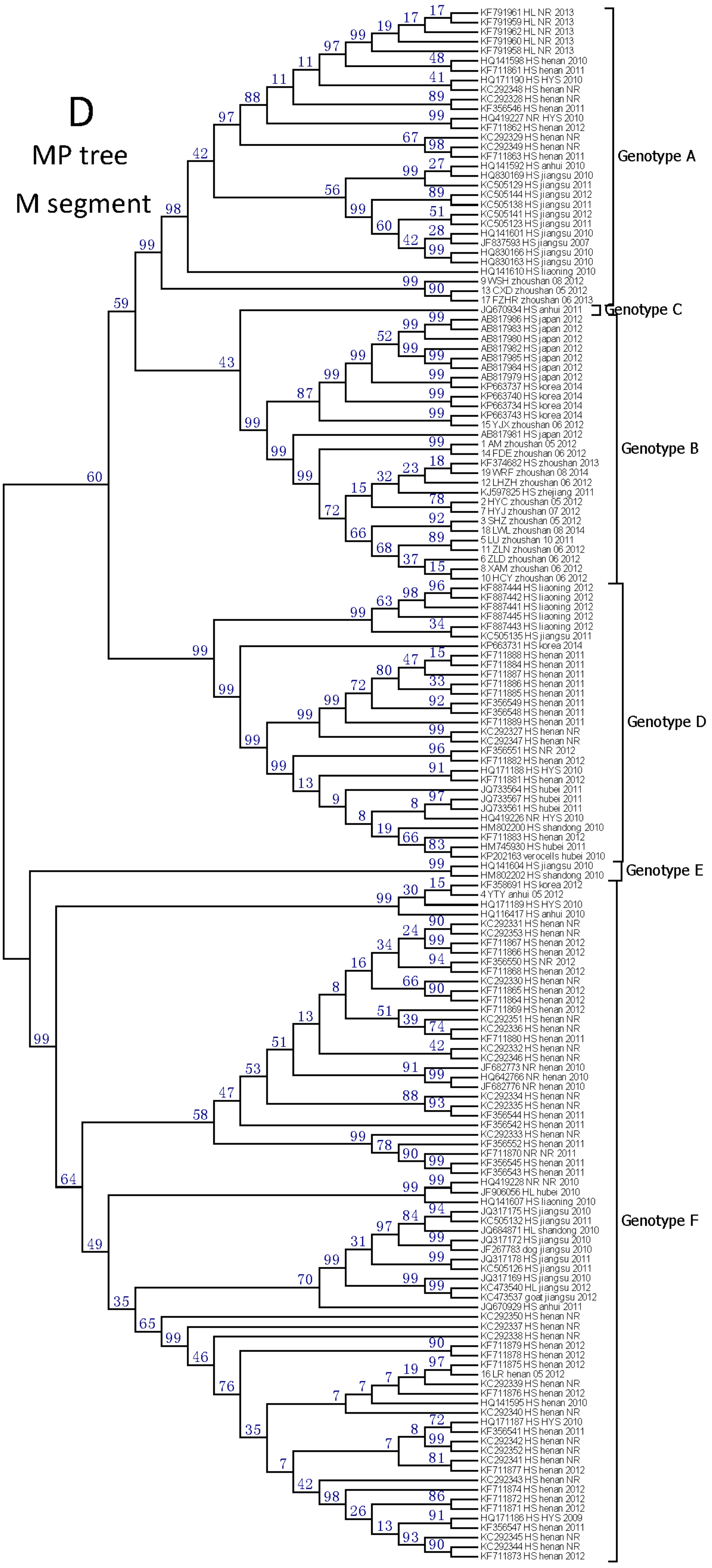
D
MP tree
M segment
KF791961 HL NR 2013
KF791959 HL NR 2013
KF791962 HL NR 2013
KF791960 HL NR 2013
KF791958 HL NR 2013
HQ141598 HS henan 2010
KF711861 HS henan 2011
HQ171190 HS HYS 2010
KC292348 HS henan NR
KC292328 HS henan NR
KF356546 HS henan 2011
HQ419227 NR HYS 2010
KF711862 HS henan 2012
KC292329 HS henan NR
KC292349 HS henan NR
KF711863 HS henan 2011
HQ141592 HS anhui 2010
HQ830169 HS jiangsu 2010
KC505129 HS jiangsu 2011
KC505144 HS jiangsu 2012
KC505138 HS jiangsu 2011
KC505141 HS jiangsu 2012
KC505123 HS jiangsu 2011
HQ141601 HS jiangsu 2010
JF837593 HS jiangsu 2007
HQ830166 HS jiangsu 2010
HQ830163 HS jiangsu 2010
HQ141610 HS liaoning 2010
9 WSH zhoushan 08 2012
13 CXD zhoushan 05 2012
17 FZHR zhoushan 06 2013
JQ670934 HS anhui 2011
AB817986 HS japan 2012
AB817983 HS japan 2012
AB817980 HS japan 2012
AB817982 HS japan 2012
AB817985 HS japan 2012
AB817984 HS japan 2012
AB817979 HS japan 2012
KP663737 HS korea 2014
KP663740 HS korea 2014
KP663734 HS korea 2014
KP663743 HS korea 2014
15 YJX zhoushan 06 2012
AB817981 HS japan 2012
1 AM zhoushan 05 2012
14 FDE zhoushan 06 2012
KF374682 HS zhoushan 2013
19 WRF zhoushan 08 2014
12 LHZH zhoushan 06 2012
KJ597825 HS zhejiang 2011
2 HYC zhoushan 05 2012
7 HYJ zhoushan 07 2012
3 SHZ zhoushan 05 2012
18 LWL zhoushan 08 2014
5 LU zhoushan 10 2011
11 ZLN zhoushan 06 2012
6 ZLD zhoushan 06 2012
8 XAM zhoushan 06 2012
10 HCY zhoushan 06 2012
KF887444 HS liaoning 2012
KF887442 HS liaoning 2012
KF887441 HS liaoning 2012
KF887445 HS liaoning 2012
KF887443 HS liaoning 2012
KC505135 HS jiangsu 2011
KP663731 HS korea 2014
KF711888 HS henan 2011
KF711884 HS henan 2011
KF711887 HS henan 2011
KF711886 HS henan 2011
KF711885 HS henan 2011
KF356549 HS henan 2011
KF356548 HS henan 2011
KF711889 HS henan 2011
KC292327 HS henan NR
KC292347 HS henan NR
KF356551 HS NR 2012
KF711882 HS henan 2012
HQ171188 HS HYS 2010
KF711881 HS henan 2012
JQ733564 HS hubei 2011
JQ733567 HS hubei 2011
JQ733561 HS hubei 2011
HQ419226 NR HYS 2010
HM802200 HS shandong 2010
KF711883 HS henan 2012
HM745930 HS hubei 2011
KP202163 verocells hubei 2010
HQ141604 HS jiangsu 2010
HM802202 HS shandong 2010
KF358691 HS korea 2012
4 YTY anhui 05 2012
HQ171189 HS HYS 2010
HQ116417 HS anhui 2010
KC292331 HS henan NR
KC292353 HS henan NR
KF711867 HS henan 2012
KF711866 HS henan 2012
KF356550 HS NR 2012
KF711868 HS henan 2012
KC292330 HS henan NR
KF711865 HS henan 2012
KF711864 HS henan 2012
KF711869 HS henan 2012
KC292351 HS henan NR
KC292336 HS henan NR
KF711880 HS henan 2011
KC292332 HS henan NR
KC292346 HS henan NR
JF682773 NR henan 2010
HQ642766 NR henan 2010
JF682776 NR henan 2010
KC292334 HS henan NR
KC292335 HS henan NR
KF356544 HS henan 2011
KF356542 HS henan 2011
KC292333 HS henan NR
KF356552 HS henan 2011
KF711870 NR NR 2011
KF356545 HS henan 2011
KF356543 HS henan 2011
HQ419228 NR NR 2010
JF906056 HL hubei 2010
HQ141607 HS liaoning 2010
JQ317175 HS jiangsu 2010
KC505132 HS jiangsu 2011
JQ684871 HL shandong 2010
JQ317172 HS jiangsu 2010
JF267783 dog jiangsu 2010
JQ317178 HS jiangsu 2011
KC505126 HS jiangsu 2011
JQ317169 HS jiangsu 2010
KC473540 HL jiangsu 2012
KC473537 goat jiangsu 2012
JQ670929 HS anhui 2011
KC292350 HS henan NR
KC292337 HS henan NR
KC292338 HS henan NR
KF711879 HS henan 2012
KF711878 HS henan 2012
KF711875 HS henan 2012
16 LR henan 05 2012
KC292339 HS henan NR
KF711876 HS henan 2012
HQ141595 HS henan 2010
KC292340 HS henan NR
HQ171187 HS HYS 2010
KF356541 HS henan 2011
KC292342 HS henan NR
KC292352 HS henan NR
KC292341 HS henan NR
KF711877 HS henan 2012
KC292343 HS henan NR
KF711874 HS henan 2012
KF711872 HS henan 2012
KF711871 HS henan 2012
HQ171186 HS HYS 2009
KF356547 HS henan 2011
KC292345 HS henan NR
KC292344 HS henan NR
KF711873 HS henan 2012
Genotype A
Genotype C
Genotype B
Genotype D
Genotype E
Genotype F

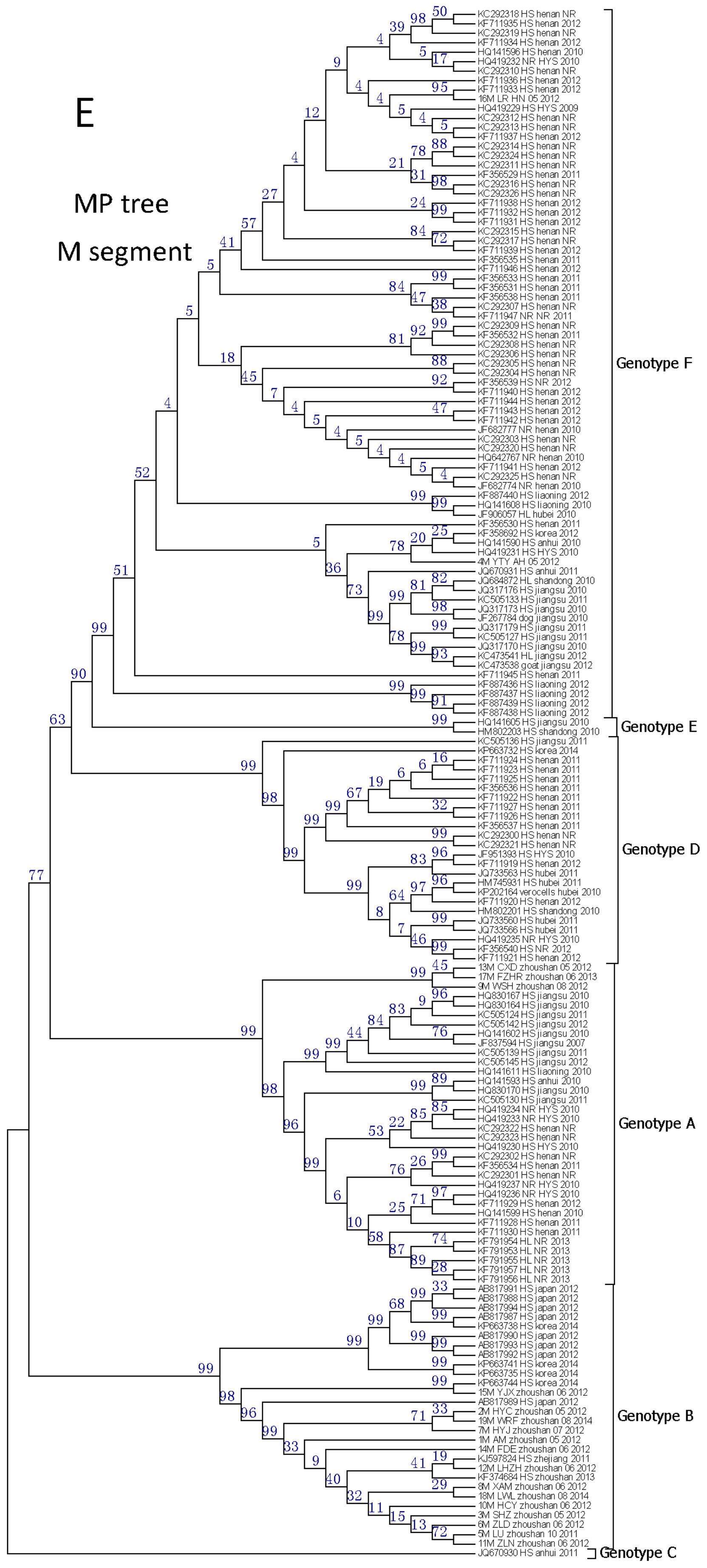

E
MP tree
M segment
KC292318 HS henan NR
KF711935 HS henan 2012
KC292319 HS henan NR
KF711934 HS henan 2012
HQ141596 HS henan 2010
HQ419232 NR HYS 2010
KC292310 HS henan NR
KF711936 HS henan 2012
KF711933 HS henan 2012
16M LR HN 05 2012
HQ419229 HS HYS 2009
KC292312 HS henan NR
KC292313 HS henan NR
KF711937 HS henan 2012
KC292314 HS henan NR
KC292324 HS henan NR
KC292311 HS henan NR
KF356529 HS henan 2011
KC292316 HS henan NR
KC292326 HS henan NR
KF711938 HS henan 2012
KF711932 HS henan 2012
KF711931 HS henan 2012
KC292315 HS henan NR
KC292317 HS henan NR
KF711939 HS henan 2012
KF356535 HS henan 2011
KF711946 HS henan 2012
KF356533 HS henan 2011
KF356531 HS henan 2011
KF356538 HS henan 2011
KC292307 HS henan NR
KF711947 NR NR 2011
KC292309 HS henan NR
KF356532 HS henan 2011
KC292308 HS henan NR
KC292306 HS henan NR
KC292305 HS henan NR
KC292304 HS henan NR
KF356539 HS NR 2012
KF711940 HS henan 2012
KF711944 HS henan 2012
KF711943 HS henan 2012
KF711942 HS henan 2012
JF682777 NR henan 2010
KC292303 HS henan NR
KC292320 HS henan NR
HQ642767 NR henan 2010
KF711941 HS henan 2012
KC292325 HS henan NR
JF682774 NR henan 2010
KF887440 HS liaoning 2012
HQ141608 HS liaoning 2010
JF906057 HL hubei 2010
KF356530 HS henan 2011
KF358692 HS korea 2012
HQ141590 HS anhui 2010
HQ419231 HS HYS 2010
4M YTY AH 05 2012
JQ670931 HS anhui 2011
JQ684872 HL shandong 2010
JQ317176 HS jiangsu 2010
KC505133 HS jiangsu 2011
JQ317173 HS jiangsu 2010
JF267784 dog jiangsu 2010
JQ317179 HS jiangsu 2011
KC505127 HS jiangsu 2011
JQ317170 HS jiangsu 2010
KC473541 HL jiangsu 2012
KC473538 goat jiangsu 2012
KF711945 HS henan 2011
KF887436 HS liaoning 2012
KF887437 HS liaoning 2012
KF887439 HS liaoning 2012
KF887438 HS liaoning 2012
HQ141605 HS jiangsu 2010
HM802203 HS shandong 2010
KC505136 HS jiangsu 2011
KP663732 HS korea 2014
KF711924 HS henan 2011
KF711923 HS henan 2011
KF711925 HS henan 2011
KF356536 HS henan 2011
KF711922 HS henan 2011
KF711927 HS henan 2011
KF711926 HS henan 2011
KF356537 HS henan 2011
KC292300 HS henan NR
KC292321 HS henan NR
JF951393 HS HYS 2010
KF711919 HS henan 2012
JQ733563 HS hubei 2011
HM745931 HS hubei 2011
KP202164 verocells hubei 2010
KF711920 HS henan 2012
HM802201 HS shandong 2010
JQ733560 HS hubei 2011
JQ733566 HS hubei 2011
HQ419235 NR HYS 2010
KF356540 HS NR 2012
KF711921 HS henan 2012
13M CXD zhoushan 05 2012
17M FZHR zhoushan 06 2013
9M WSH zhoushan 08 2012
HQ830167 HS jiangsu 2010
HQ830164 HS jiangsu 2010
KC505124 HS jiangsu 2011
KC505142 HS jiangsu 2012
HQ141602 HS jiangsu 2010
JF837594 HS jiangsu 2007
KC505139 HS jiangsu 2011
KC505145 HS jiangsu 2012
HQ141611 HS liaoning 2010
HQ141593 HS anhui 2010
HQ830170 HS jiangsu 2010
KC505130 HS jiangsu 2011
HQ419234 NR HYS 2010
HQ419233 NR HYS 2010
KC292322 HS henan NR
KC292323 HS henan NR
HQ419230 HS HYS 2010
KC292302 HS henan NR
KF356534 HS henan 2011
KC292301 HS henan NR
HQ419237 NR HYS 2010
HQ419236 NR HYS 2010
KF711929 HS henan 2012
HQ141599 HS henan 2010
KF711928 HS henan 2011
KF711930 HS henan 2011
KF791954 HL NR 2013
KF791953 HL NR 2013
KF791955 HL NR 2013
KF791957 HL NR 2013
KF791956 HL NR 2013
AB817991 HS japan 2012
AB817988 HS japan 2012
AB817994 HS japan 2012
AB817987 HS japan 2012
KP663738 HS korea 2014
AB817990 HS japan 2012
AB817993 HS japan 2012
AB817992 HS japan 2012
KP663741 HS korea 2014
KP663735 HS korea 2014
KP663744 HS korea 2014
15M YJX zhoushan 06 2012
AB817989 HS japan 2012
2M HYC zhoushan 05 2012
19M WRF zhoushan 08 2014
7M HYJ zhoushan 07 2012
1M AM zhoushan 05 2012
14M FDE zhoushan 06 2012
KJ597824 HS zhejiang 2011
12M LHZH zhoushan 06 2012
KF374684 HS zhoushan 2013
8M XAM zhoushan 06 2012
18M LWL zhoushan 08 2014
10M HCY zhoushan 06 2012
3M SHZ zhoushan 05 2012
6M ZLD zhoushan 06 2012
5M LU zhoushan 10 2011
11M ZLN zhoushan 06 2012
JQ670930 HS anhui 2011
Genotype F
Genotype E
Genotype D
Genotype A
Genotype B
Genotype C

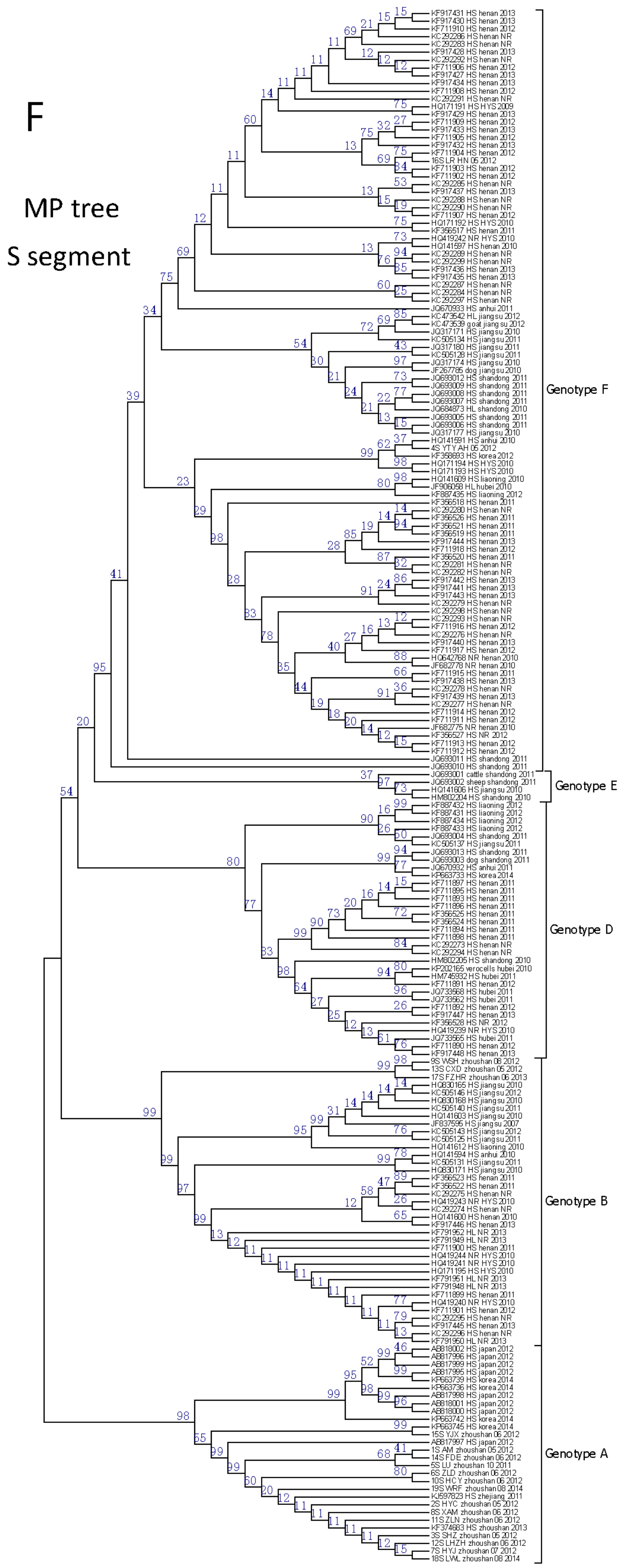

Supplementary Figure S1. Phylogenetic trees of L, M, S genomic segments using NJ and MP methods. A-C: NJ trees; D-F: MP trees.

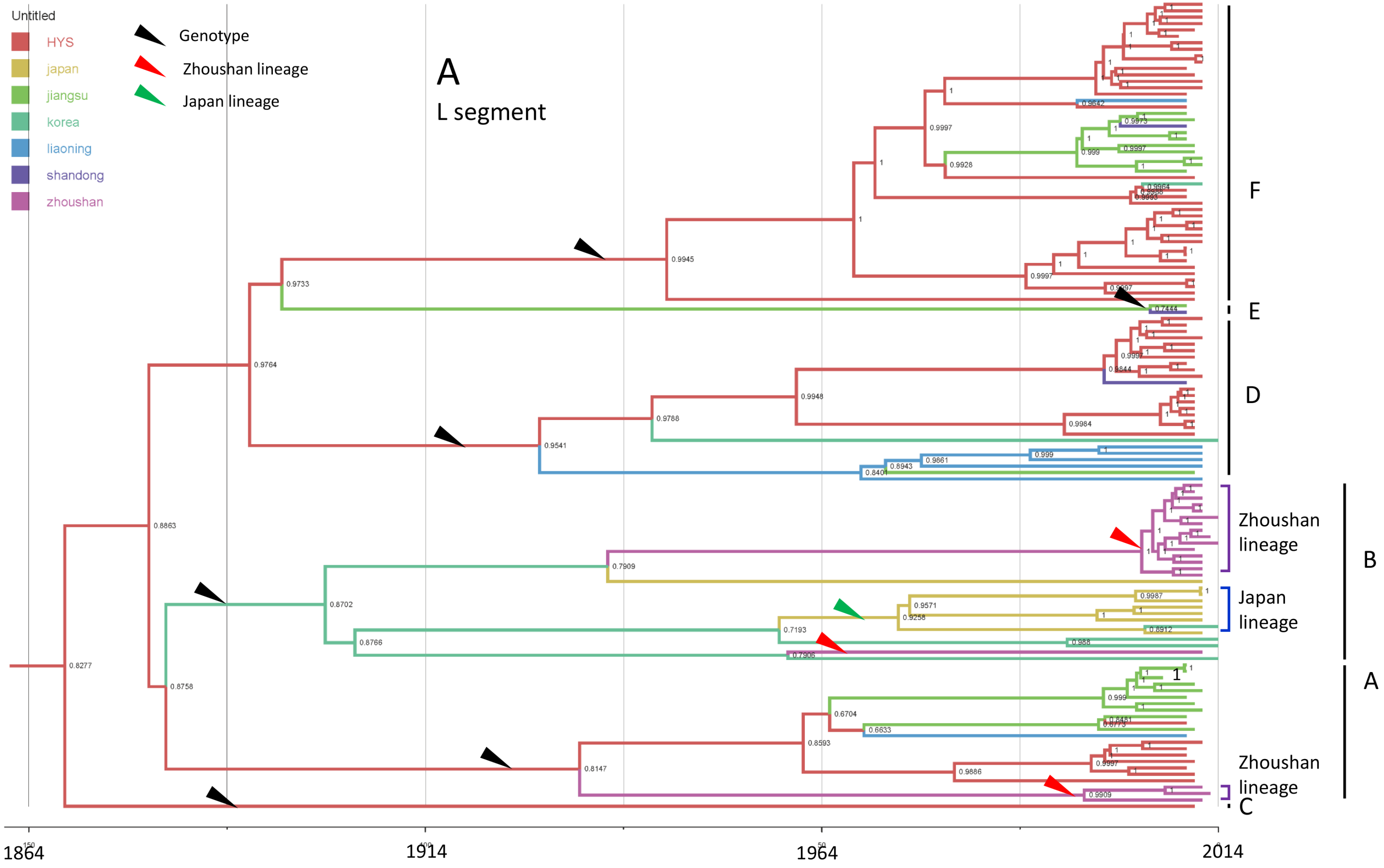
Untitled
HYS
japan
jiangsu
korea
liaoning
shandong
zhoushan
Genotype
Zhoushan lineage
Japan lineage
A
L segment
F
E
D
Zhoushan lineage
Japan lineage
B
A
Zhoushan lineage
C
1864
1914
1964
2014

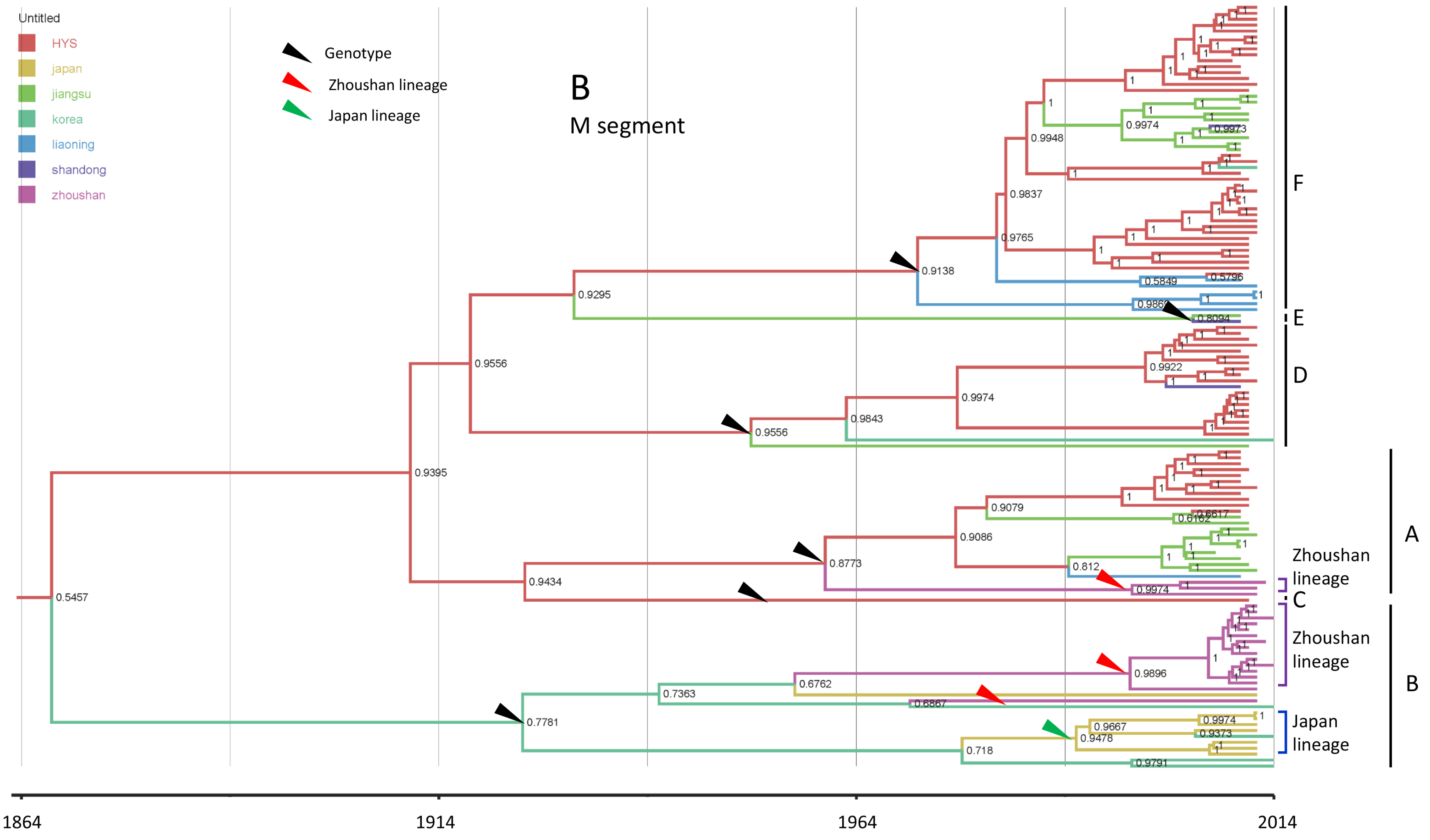
Untitled
HYS
japan
jiangsu
korea
liaoning
shandong
zhoushan
Genotype
Zhoushan lineage
Japan lineage
B
M segment
F
E
D
A
Zhoushan lineage
C
Zhoushan lineage
B
Japan lineage
1864
1914
1964
2014

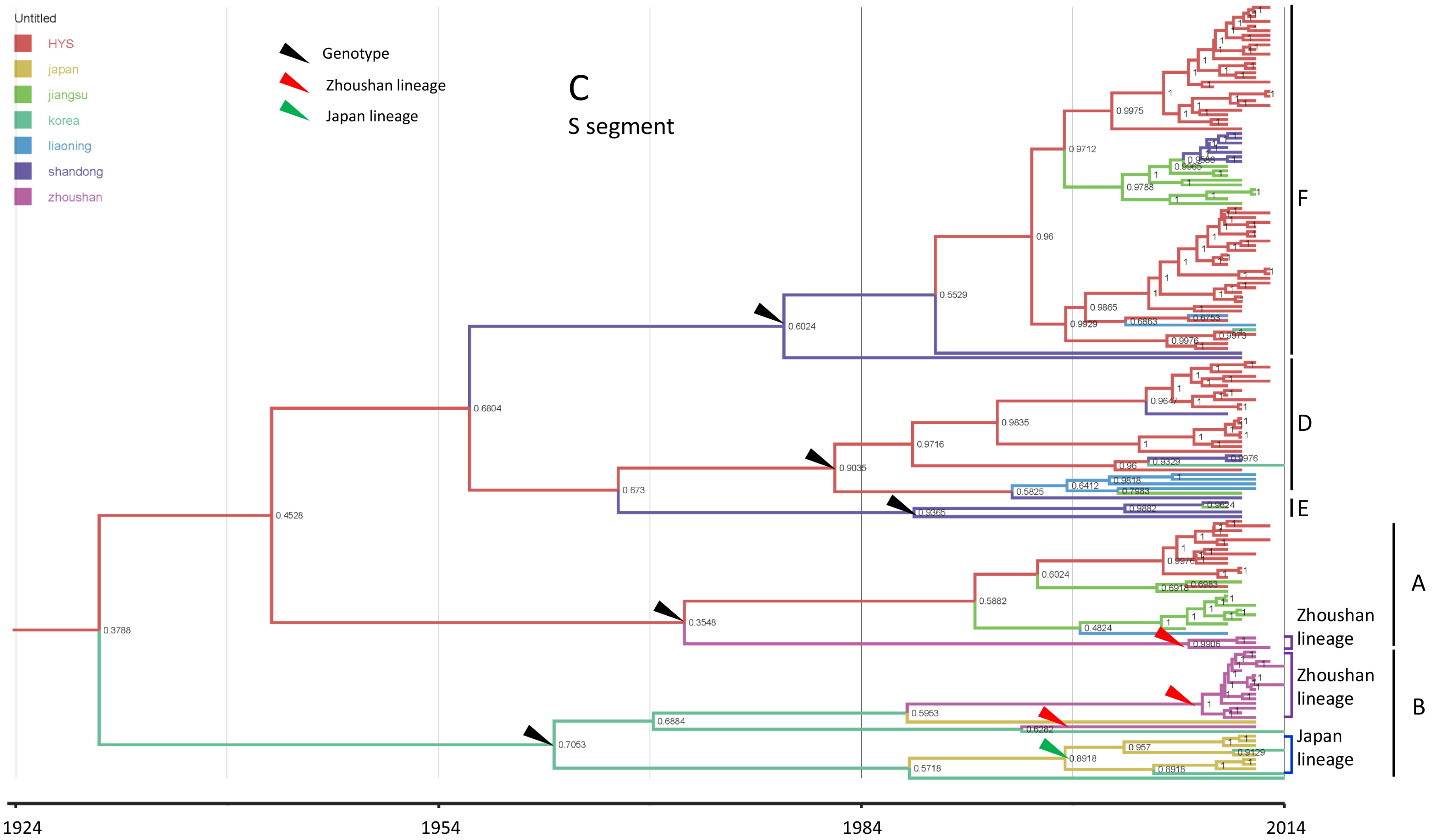

Supplementary Figure S2. Maximum clade credibility (MCC) trees for of SFTSV L (A), M (B), and S (C) segments.

Supplementary Table S1. Comparison of clinical phenotypes and clinical laboratory parameters of SFTS patients between Zhoushan and other regions/countries.

	Zhoushan (n =21)	Mainland of China(*n=311*)	Mainland of China (*n=81*)	Japan (*n* =11)
Clinical phenotype				
Fever	21	311	81	11
Diarrhea	7	80	34	7
Weakness	5	297 *	Unknown	Unknown
Cough	2	Unknown	8	1
Abdominal distension	1	Unknown	40 *	6 *
Restless	1	Unknown	Unknown	Unknown
Arthralgia	1	Unknown	Unknown	1
Myalgia	1	Unknown	22	1
Leukopenia (leukocyte count, < 4.0 × 10^9 leukocytes/L)	20	Unknown	64	11
Thrombocytopenia (platelet count, < 100 × 10^9 platelets/L)	21	Unknown	73	11
Clinical laboratory parameter				
WBC (10^9/L)	1.5±0.8	2.9±2.2	Unknown	Unknown
PLT (10^9/L)	33±17	66±33	Unknown	Unknown
HGB (g/L)	108±23	128±20	Unknown	Unknown
AST (U/L)	28 (50-1201)	101 (12–1739)	Unknown	Unknown
ALT (U/L)	125 (56-516)	65 (10–800)	Unknown	Unknown
CK (U/L)	1129 (122-8495)	373 (26–7000)	Unknown	Unknown
LDH (U/L)	946 (266-2415)	473 (14–3354)	Unknown	Unknown
Reference	This study	[15]	[1]	[12]

Statistic analyses use. * means p value <0.05.

* P<0.05 (χ^2 test).

Supplementary Table S2. Sequence information of SFTSV strains used in this study.

Sample location	Viral strain	Host	Sample time	S segment		M segment		L segment		Genotype of strain	Sequence source
				Accession number	Genotype	Accession number	Genotype	Accession number	Genotype		
Anhui	AH15	Homo sapiens	2010.8	HQ141594	A	HQ141593	A	HQ141592	A	A	GenBank
Henan	HN13	Homo sapiens	2010.7	HQ141600	A	HQ141599	A	HQ141598	A	A	GenBank
Henan	HNXY_188	Homo sapiens	NA	KC292274	A	KC292301	A	KC292328	A	A	GenBank
Henan	HNXY_206	Homo sapiens	NA	KC292275	A	KC292302	A	KC292329	A	A	GenBank
Henan	HNXY_278	Homo sapiens	NA	KC292295	A	KC292322	A	KC292348	A	A	GenBank
Henan	HNXY_191	Homo sapiens	NA	KC292296	A	KC292323	A	KC292349	A	A	GenBank
Henan, Xinyang	YXX2	Homo sapiens	2011.5	KF356522	A	KF356534	A	KF356546	A	A	GenBank
Henan, Xinyang guangshan	2011YSH52	Homo sapiens	2011.8	KF711900	A	KF711928	A	KF711861	A	A	GenBank
Henan, Xinyang xinxian	2012YXX1	Homo sapiens	2012.8	KF711901	A	KF711929	A	KF711862	A	A	GenBank
Huaiyangshan	2010-FQM	NA	2010	HQ419240	A	HQ419236	A	HQ419227	A	A	GenBank
Huaiyangshan	S-WJQ	Homo sapiens	2010.5	HQ171195	A	HQ419230	A	HQ171190	A	A	GenBank
Jiangsu	JS2007-01	Homo sapiens	2007	JF837595	A	JF837594	A	JF837593	A	A	GenBank
Jiangsu	JS2011-004	Homo sapiens	2011	KC505125	A	KC505124	A	KC505123	A	A	GenBank
Jiangsu	JS2011-013-1	Homo sapiens	2011	KC505128	F	KC505127	F	KC505126	F	A	GenBank

Jiangsu	JS2011-027	Homo sapiens	2011	KC505131	A	KC505130	A	KC505129	A	A	GenBank
Jiangsu	JS2011-109	Homo sapiens	2011	KC505140	A	KC505139	A	KC505138	A	A	GenBank
Jiangsu	JS3	Homo sapiens	2010.8	HQ141603	A	HQ141602	A	HQ141601	A	A	GenBank
Jiangsu	JS6	Homo sapiens	2010.10	HQ830171	A	HQ830170	A	HQ830169	A	A	GenBank
Jiangsu	JS24	Homo sapiens	2010.11	HQ830165	A	HQ830164	A	HQ830163	A	A	GenBank
Jiangsu	JS26	Homo sapiens	2010.11	HQ830168	A	HQ830167	A	HQ830166	A	A	GenBank
Jiangsu	JS2012-020	Homo sapiens	2012.3	KC505143	A	KC505142	A	KC505141	A	A	GenBank
Jiangsu	JS2012-035	Homo sapiens	2012.3	KC505146	A	KC505145	A	KC505144	A	A	GenBank
Liaoning	LN3/China/2010	Homo sapiens	2010.7	HQ141612	A	HQ141611	A	HQ141610	A	A	GenBank
NA	HL/Injected	Homo sapiens	2013.9	KF791948	A	KF791953	A	KF791958	A	A	GenBank
NA	HL/Egg/G2	Haemaphysalis longicornis	2013.9	KF791949	A	KF791954	A	KF791959	A	A	GenBank
NA	HL/Larvae/G2	Haemaphysalis longicornis	2013.9	KF791950	A	KF791955	A	KF791960	A	A	GenBank
NA	HL/Nymph/G2	Haemaphysalis longicornis	2013.9	KF791951	A	KF791956	A	KF791961	A	A	GenBank
NA	HL/Adult/G2	Haemaphysalis longicornis	2013.9	KF791952	A	KF791957	A	KF791962	A	A	GenBank
Zhoushan	ZJZHSH-CXD/China/	Homo sapiens	2012.5	KR017820	A	KR017858	A	KR017839	A	A	This study

	05/2012									
Zhoushan	ZJZHSH-WSH/China/ 08/2012	Homo sapiens	2012.8	KR017816	A	KR017854	A	KR017835	A	A	This study
Zhoushan	ZJZHSH-FZHR/China /07/2013	Homo sapiens	2013.7	KR017824	A	KR017862	A	KR017843	A	A	This study
Henan, Xinyang guangshan	2011YSC60*	Homo sapiens	2011.8	KF711899	A	KF711945	F	KF711863	A	AFA	GenBank
Japan	SPL035A	Homo sapiens	2012	AB818002	B	AB817994	B	AB817986	B	B	GenBank
Japan	YG1	Homo sapiens	2012	AB817995	B	AB817987	B	AB817979	B	B	GenBank
Japan	SPL003A	Homo sapiens	2012	AB817996	B	AB817988	B	AB817980	B	B	GenBank
Japan	SPL004A	Homo sapiens	2012	AB817997	B	AB817989	B	AB817981	B	B	GenBank
Japan	SPL005A	Homo sapiens	2012	AB817998	B	AB817990	B	AB817982	B	B	GenBank
Japan	SPL010A	Homo sapiens	2012	AB817999	B	AB817991	B	AB817983	B	B	GenBank
Japan	SPL030A	Homo sapiens	2012	AB818000	B	AB817992	B	AB817984	B	B	GenBank
Japan	SPL032A	Homo sapiens	2012	AB818001	B	AB817993	B	AB817985	B	B	GenBank
South Korea	KAGWH3	Homo sapiens	2014.9	KP663736	B	KP663735	B	KP663734	B	B	GenBank
South Korea	KAGBH5	Homo sapiens	2014.9	KP663739	B	KP663738	B	KP663737	B	B	GenBank
South Korea	KAGBH6	Homo sapiens	2014.9	KP663742	B	KP663741	B	KP663740	B	B	GenBank
South Korea	KACNH3	Homo sapiens	2014.9	KP663745	B	KP663744	B	KP663743	B	B	GenBank

Zhejiang(zhoushan)	Zhejiang/01/2011	Homo sapiens	2011.6	KJ597823	B	KJ597824	B	KJ597825	B	B	GenBank
Zhoushan	Zhao	Homo sapiens	2013.3	KF374683	B	KF374684	B	KF374682	B	B	GenBank
Zhoushan	ZJZHSH-LU/China/10/2011	Homo sapiens	2011.10	KR017812	B	KR017850	B	KR017831	B	B	This study
Zhoushan	ZJZHSH-AM/China/05/2012	Homo sapiens	2012.5	KR017808	B	KR017846	B	KR017827	B	B	This study
Zhoushan	ZJZHSH-HYC/China/05/2012	Homo sapiens	2012.5	KR017809	B	KR017847	B	KR017828	B	B	This study
Zhoushan	ZJZHSH-SHZ/China/05/2012	Homo sapiens	2012.5	KR017810	B	KR017848	B	KR017829	B	B	This study
Zhoushan	ZJZHSH-XAM/China/06/2012	Homo sapiens	2012.6	KR017815	B	KR017853	B	KR017834	B	B	This study
Zhoushan	ZJZHSH-FDE/China/06/2012	Homo sapiens	2012.6	KR017821	B	KR017859	B	KR017840	B	B	This study
Zhoushan	ZJZHSH-HCY/China/06/2012	Homo sapiens	2012.6	KR017817	B	KR017855	B	KR017836	B	B	This study
Zhoushan	ZJZHSH-ZLN/China/06/2012	Homo sapiens	2012.6	KR017818	B	KR017856	B	KR017837	B	B	This study

Zhoushan	ZJZHSH-LHZH/China/06/2012	Homo sapiens	2012.6	KR017819	B	KR017857	B	KR017838	B	B	This study
Zhoushan	ZJZHSH-ZLD/China/06/2012	Homo sapiens	2012.6	KR017813	B	KR017851	B	KR017832	B	B	This study
Zhoushan	ZJZHSH-YJX/China/06/2012	Homo sapiens	2012.6	KR017822	B	KR017860	B	KR017841	B	B	This study
Zhoushan	ZJZHSH-HYJ/China/07/2012	Homo sapiens	2012.7	KR017814	B	KR017852	B	KR017833	B	B	This study
Zhoushan	ZJZHSH-WRF/China/08/2014	Homo sapiens	2014.8	KR017826	B	KR017864	B	KR017845	B	B	This study
Zhoushan	ZJZHSH-LWL/China/08/2014	Homo sapiens	2014.8	KR017825	B	KR017863	B	KR017844	B	B	This study
Anhui	AHL/China/2011*	Homo sapiens	2011.6	JQ670932	D	JQ670930	C	JQ670934	C	CCD	GenBank
Henan	HNXY_212	Homo sapiens	NA	KC292273	D	KC292300	D	KC292327	D	D	GenBank
Henan	HNXY_327	Homo sapiens	NA	KC292294	D	KC292321	D	KC292347	D	D	GenBank
Henan, Xinyang	YSHX002	Homo sapiens	2012	KF356528	D	KF356540	D	KF356551	D	D	GenBank
Henan, Xinyang	YGS1	Homo sapiens	2011.4	KF356525	D	KF356537	D	KF356549	D	D	GenBank
Henan, Xinyang	YSC19	Homo sapiens	2011.5	KF356524	D	KF356536	D	KF356548	D	D	GenBank
Henan, Xinyang	2011YGS5	Homo sapiens	2011.8	KF711893	D	KF711925	D	KF711884	D	D	GenBank

guangshan											
Henan, Xinyang guangshan	2011YSC22	Homo sapiens	2011.8	KF711894	D	KF711927	D	KF711888	D	D	GenBank
Henan, Xinyang guangshan	2011YGS7	Homo sapiens	2011.8	KF711895	D	KF711922	D	KF711885	D	D	GenBank
Henan, Xinyang pingqiao	2011YPQ17	Homo sapiens	2011.8	KF711896	D	KF711923	D	KF711887	D	D	GenBank
Henan, Xinyang pingqiao	2011YPQ11	Homo sapiens	2011.8	KF711897	D	KF711926	D	KF711886	D	D	GenBank
Henan, Xinyang shihe	2012YSH91	Homo sapiens	2012.8	KF711891	D	KF711920	D	KF711883	D	D	GenBank
Henan, Xinyang xinxian	2011YXX9	Homo sapiens	2011.8	KF711898	D	KF711924	D	KF711889	D	D	GenBank
Henan, Xinyang xinxian	2012YSH9	Homo sapiens	2012.8	KF711890	D	KF711919	D	KF711881	D	D	GenBank
Henan, Xinyang xinxian	2012YSH6	Homo sapiens	2012.8	KF711892	D	KF711921	D	KF711882	D	D	GenBank
Huaiyangshan	2010-WSQ	NA	2010	HQ419239	D	HQ419235	D	HQ419226	D	D	GenBank
Hubei	HB29	Vero cells	2010	KP202165	D	KP202164	D	KP202163	D	D	GenBank

Hubei	HB29	Homo sapiens	2010.5	HM745932	D	HM745931	D	HM745930	D	D	GenBank
Hubei	HB154/China/2011	Homo sapiens	2011.8	JQ733562	D	JQ733560	D	JQ733561	D	D	GenBank
Hubei	HB155/China/2011	Homo sapiens	2011.8	JQ733565	D	JQ733563	D	JQ733564	D	D	GenBank
Hubei	HB156/China/2011	Homo sapiens	2011.8	JQ733568	D	JQ733566	D	JQ733567	D	D	GenBank
Jiangsu	JS2011-062	Homo sapiens	2011	KC505137	D	KC505136	D	KC505135	D	D	GenBank
Shandong	SD24	Homo sapiens	2010	HM802205	D	HM802201	D	HM802200	D	D	GenBank
South Korea	KASJH	Homo sapiens	2014.9	KP663733	D	KP663732	D	KP663731	D	D	GenBank
Huaiyangshan	S-HZM*	Homo sapiens	2010.5	HQ171193	F	JF951393	D	HQ171188	D	DDF	GenBank
Liaoning	LN2012-14*	Homo sapiens	2012.7	KF887431	D	KF887436	F	KF887441	D	DFD	GenBank
Liaoning	LN2012-34*	Homo sapiens	2012.7	KF887432	D	KF887437	F	KF887442	D	DFD	GenBank
Liaoning	LN2012-42*	Homo sapiens	2012.8	KF887434	D	KF887439	F	KF887444	D	DFD	GenBank
Liaoning	LN2012-41*	Homo sapiens	2012.8	KF887433	D	KF887438	F	KF887443	D	DFD	GenBank
Liaoning	LN2012-58*	Homo sapiens	2012.8	KF887435	F	KF887440	F	KF887445	D	DFF	GenBank
Jiangsu	JS4	Homo sapiens	2010.8	HQ141606	E	HQ141605	E	HQ141604	E	E	GenBank
Shandong	SD4	Homo sapiens	2010	HM802204	E	HM802203	E	HM802202	E	E	GenBank
Anhui	AH12	Homo sapiens	2010.8	HQ141591	F	HQ141590	F	HQ116417	F	F	GenBank
Anhui	AHZ/China/2011	Homo sapiens	2011.7	JQ670933	F	JQ670931	F	JQ670929	F	F	GenBank
Anhui	AH-YTY/China/05/2012	Homo sapiens	2012.5	KR017811	F	KR017849	F	KR017830	F	F	GenBank

Henan	BX-2010/Henan/CHN	NA	2010	HQ642768	F	HQ642767	F	HQ642766	F	F	GenBank
Henan	BX-2010/Henan/CHN isolate 20	NA	2010	JF682775	F	JF682774	F	JF682773	F	F	GenBank
Henan	BX-2010/Henan/CHN 69	NA	2010	JF682778	F	JF682777	F	JF682776	F	F	GenBank
Henan	HN6	Homo sapiens	2010.7	HQ141597	F	HQ141596	F	HQ141595	F	F	GenBank
Henan	HNXY_293	Homo sapiens	NA	KC292276	F	KC292303	F	KC292330	F	F	GenBank
Henan	HNXY_170	Homo sapiens	NA	KC292277	F	KC292304	F	KC292353	F	F	GenBank
Henan	HNXY_157	Homo sapiens	NA	KC292278	F	KC292305	F	KC292331	F	F	GenBank
Henan	HNXY_174	Homo sapiens	NA	KC292279	F	KC292306	F	KC292332	F	F	GenBank
Henan	HNXY_144	Homo sapiens	NA	KC292280	F	KC292307	F	KC292333	F	F	GenBank
Henan	HNXY_186	Homo sapiens	NA	KC292281	F	KC292308	F	KC292334	F	F	GenBank
Henan	HNXY_262	Homo sapiens	NA	KC292282	F	KC292309	F	KC292335	F	F	GenBank
Henan	HNXY_31	Homo sapiens	NA	KC292283	F	KC292310	F	KC292336	F	F	GenBank
Henan	HNXY_93	Homo sapiens	NA	KC292284	F	KC292311	F	KC292337	F	F	GenBank
Henan	HNXY_115	Homo sapiens	NA	KC292285	F	KC292312	F	KC292338	F	F	GenBank
Henan	HNXY_231	Homo sapiens	NA	KC292286	F	KC292313	F	KC292339	F	F	GenBank
Henan	HNXY_164	Homo sapiens	NA	KC292287	F	KC292314	F	KC292340	F	F	GenBank
Henan	HNXY_207	Homo sapiens	NA	KC292288	F	KC292315	F	KC292341	F	F	GenBank

Henan	HNXY_130	Homo sapiens	NA	KC292289	F	KC292316	F	KC292342	F	F	GenBank
Henan	HNXY_224	Homo sapiens	NA	KC292290	F	KC292317	F	KC292343	F	F	GenBank
Henan	HNXY_2	Homo sapiens	NA	KC292291	F	KC292318	F	KC292344	F	F	GenBank
Henan	HNXY_319	Homo sapiens	NA	KC292292	F	KC292319	F	KC292345	F	F	GenBank
Henan	HNXY_245	Homo sapiens	NA	KC292293	F	KC292320	F	KC292346	F	F	GenBank
Henan	HNXY_182	Homo sapiens	NA	KC292297	F	KC292324	F	KC292350	F	F	GenBank
Henan	HNXY_202	Homo sapiens	NA	KC292298	F	KC292325	F	KC292351	F	F	GenBank
Henan	HNXY_195	Homo sapiens	NA	KC292299	F	KC292326	F	KC292352	F	F	GenBank
Henan	HN-LR/China/05/2012	Homo sapiens	2012.5	KR017823	F	KR017861	F	KR017842	F	F	This study
Henan, Nanyang	YNY1	Homo sapiens	2011.7	KF356526	F	KF356538	F	KF356552	F	F	GenBank
Henan, Xinyang	YPQX03	Homo sapiens	2012	KF356527	F	KF356539	F	KF356550	F	F	GenBank
Henan, Xinyang	YPQ2	Homo sapiens	2011.4	KF356519	F	KF356531	F	KF356543	F	F	GenBank
Henan, Xinyang	YPQ5	Homo sapiens	2011.5	KF356520	F	KF356532	F	KF356544	F	F	GenBank
Henan, Xinyang	YXX1	Homo sapiens	2011.5	KF356521	F	KF356533	F	KF356545	F	F	GenBank
Henan, Xinyang	YSH39	Homo sapiens	2011.6	KF356517	F	KF356529	F	KF356541	F	F	GenBank
Henan, Xinyang	YPQ133	Homo sapiens	2011.7	KF356518	F	KF356530	F	KF356542	F	F	GenBank
Henan, Xinyang guangshan	2012YGS4	Homo sapiens	2012.8	KF711904	F	KF711933	F	KF711875	F	F	GenBank
Henan, Xinyang	2012YSH37	Homo sapiens	2012.8	KF711905	F	KF711936	F	KF711871	F	F	GenBank

guangshan										
Henan, Xinyang guangshan	2012YGS10	Homo sapiens	2012.8	KF711909	F	KF711937	F	KF711872	F	F	GenBank
Henan, Xinyang gushi	2012YSH86	Homo sapiens	2012.8	KF711907	F	KF711939	F	KF711877	F	F	GenBank
Henan, Xinyang pingqiao	2012YSH10	Homo sapiens	2012.8	KF711911	F	KF711940	F	KF711868	F	F	GenBank
Henan, Xinyang shangcheng	2012YSC6	Homo sapiens	2012.8	KF711902	F	KF711931	F	KF711879	F	F	GenBank
Henan, Xinyang shangcheng	2012YSH89	Homo sapiens	2012.8	KF711906	F	KF711935	F	KF711873	F	F	GenBank
Henan, Xinyang shangcheng	2012YSH16	Homo sapiens	2012.8	KF711908	F	KF711934	F	KF711874	F	F	GenBank
Henan, Xinyang shangcheng	2012YSH14	Homo sapiens	2012.8	KF711910	F	KF711938	F	KF711876	F	F	GenBank
Henan, Xinyang shangcheng	2012YSH104	Homo sapiens	2012.8	KF711912	F	KF711943	F	KF711867	F	F	GenBank
Henan, Xinyang shihe	2012YSH93	Homo sapiens	2012.8	KF711913	F	KF711946	F	KF711866	F	F	GenBank

Henan, Xinyang shihe	2012YSH105	Homo sapiens	2012.8	KF711914	F	KF711944	F	KF711869	F	F	GenBank
Henan, Xinyang shihe	2012YSH107	Homo sapiens	2012.8	KF711916	F	KF711942	F	KF711864	F	F	GenBank
Henan, Xinyang shihe	2012YSH92	Homo sapiens	2012.8	KF711917	F	KF711941	F	KF711865	F	F	GenBank
Henan, Xinyang shihe	2012YSH27	Homo sapiens	2012.8	KF711903	F	KF711932	F	KF711878	F	F	GenBank
Huaiyangshan	S-WJ	Homo sapiens	2009.6	HQ171191	F	HQ419229	F	HQ171186	F	F	GenBank
Huaiyangshan	S-WWG	Homo sapiens	2010.5	HQ171194	F	HQ419231	F	HQ171189	F	F	GenBank
Huaiyangshan	XCQ-A112S	Haemaphysalis longicornis	2010.5	JF906058	F	JF906057	F	JF906056	F	F	GenBank
Hubei	HB29	Homo sapiens	2010.5	NC_018137	F	NC_018138	F	NC_018136	F	F	GenBank
Jiangsu	JS2011-034	Homo sapiens	2011	KC505134	F	KC505133	F	KC505132	F	F	GenBank
Jiangsu	JS2010-014	Homo sapiens	2010.11	JQ317171	F	JQ317170	F	JQ317169	F	F	GenBank
Jiangsu	JS2010-015	Homo sapiens	2010.11	JQ317174	F	JQ317173	F	JQ317172	F	F	GenBank
Jiangsu	JS2010-018	Homo sapiens	2010.12	JQ317177	F	JQ317176	F	JQ317175	F	F	GenBank
Jiangsu	JSD1	Dog	2011.1	JF267785	F	JF267784	F	JF267783	F	F	GenBank
Jiangsu	JS2010-019	Homo sapiens	2011.1	JQ317180	F	JQ317179	F	JQ317178	F	F	GenBank

Jiangsu	JS2012-goat01	Goat	2012.9	KC473539	F	KC473538	F	KC473537	F	F	GenBank
Jiangsu	JS2012-tick01	Haemaphysalis longicornis	2012.9	KC473542	F	KC473541	F	KC473540	F	F	GenBank
Liaoning	LN2/China/2010	Homo sapiens	2010.7	HQ141609	F	HQ141608	F	HQ141607	F	F	GenBank
Shandong	SDLZtick12/2010	Haemaphysalis longicornis	2010.7	JQ684873	F	JQ684872	F	JQ684871	F	F	GenBank
South Korea	Gangwon/Korea/2012	Homo sapiens	2012.8	KF358693	F	KF358692	F	KF358691	F	F	GenBank
Henan, Xinyang pingqiao	2011YPQ12*	Homo sapiens	2011.8	KF711915	F	KF711930	A	KF711880	F	FAF	GenBank
Henan, Xinyang	YSC3*	Homo sapiens	2011.5	KF356523	A	KF356535	F	KF356547	F	FFA	GenBank
Henan, Xinyang	2012YSH45	Homo sapiens	2012.8	KF711918	F	NA	NA	NA	NA	ND	GenBank
Henan, Xinyang	2013082S	Homo sapiens	2013.10	KF917427	F	NA	NA	NA	NA	ND	GenBank
Henan, Xinyang	2013066S	Homo sapiens	2013.10	KF917428	F	NA	NA	NA	NA	ND	GenBank
Henan, Xinyang	2013067S	Homo sapiens	2013.10	KF917429	F	NA	NA	NA	NA	ND	GenBank
Henan, Xinyang	2013041S	Homo sapiens	2013.10	KF917430	F	NA	NA	NA	NA	ND	GenBank
Henan, Xinyang	2013043S	Homo sapiens	2013.10	KF917431	F	NA	NA	NA	NA	ND	GenBank
Henan, Xinyang	2013048S	Homo sapiens	2013.10	KF917432	F	NA	NA	NA	NA	ND	GenBank
Henan, Xinyang	2013075S	Homo sapiens	2013.10	KF917433	F	NA	NA	NA	NA	ND	GenBank
Henan, Xinyang	2013040S	Homo sapiens	2013.10	KF917434	F	NA	NA	NA	NA	ND	GenBank

Henan, Xinyang	2013049S	Homo sapiens	2013.10	KF917435	F	NA	NA	NA	NA	ND	GenBank
Henan, Xinyang	2013051S	Homo sapiens	2013.10	KF917436	F	NA	NA	NA	NA	ND	GenBank
Henan, Xinyang	2013037S	Homo sapiens	2013.10	KF917437	F	NA	NA	NA	NA	ND	GenBank
Henan, Xinyang	2013081S	Homo sapiens	2013.10	KF917438	F	NA	NA	NA	NA	ND	GenBank
Henan, Xinyang	2013001S	Homo sapiens	2013.10	KF917439	F	NA	NA	NA	NA	ND	GenBank
Henan, Xinyang	2013045S	Homo sapiens	2013.10	KF917440	F	NA	NA	NA	NA	ND	GenBank
Henan, Xinyang	2013077S	Homo sapiens	2013.10	KF917441	F	NA	NA	NA	NA	ND	GenBank
Henan, Xinyang	2013079S	Homo sapiens	2013.10	KF917442	F	NA	NA	NA	NA	ND	GenBank
Henan, Xinyang	2013052S	Homo sapiens	2013.10	KF917443	F	NA	NA	NA	NA	ND	GenBank
Henan, Xinyang	2013063S	Homo sapiens	2013.10	KF917444	F	NA	NA	NA	NA	ND	GenBank
Henan, Xinyang	2013038S	Homo sapiens	2013.10	KF917445	A	NA	NA	NA	NA	ND	GenBank
Henan, Xinyang	2013070S	Homo sapiens	2013.10	KF917446	A	NA	NA	NA	NA	ND	GenBank
Henan, Xinyang	2013039S	Homo sapiens	2013.10	KF917447	D	NA	NA	NA	NA	ND	GenBank
Henan, Xinyang	2013050S	Homo sapiens	2013.10	KF917448	D	NA	NA	NA	NA	ND	GenBank
Henan, Xinyang guangshan	2011YSC45	NA	2011.8	NA	NA	NA	NA	KF711870	F	ND	GenBank
Henan, Xinyang guangshan	2011YSH45	Homo sapiens	NA	NA	NA	KF711947	F	NA	NA	ND	GenBank
Huaiyangshan	2010-ZGQ	NA	2010	HQ419241	A	HQ419233	A	NA	NA	ND	GenBank

Huaiyangshan	2010-WWX	NA	2010	HQ419242	F	HQ419232	F	NA	NA	ND	GenBank
Huaiyangshan	2010-LZR	NA	2010	HQ419243	A	HQ419237	A	NA	NA	ND	GenBank
Huaiyangshan	2010-CBX	NA	2010	HQ419244	A	HQ419234	A	NA	NA	ND	GenBank
Huaiyangshan	2010-WJ	NA	2010	NA	NA	HQ419229	F	NA	NA	ND	GenBank
Huaiyangshan	2010-T112	NA	2010	NA	NA	NA	NA	HQ419228	F	ND	GenBank
Huaiyangshan	S-HGX	Homo sapiens	2010.5	HQ171192	F	NA	NA	HQ171187	F	ND	GenBank
Shandong	SDLZCattle01/2011	Cattle	2011.6	JQ693001	E	NA	NA	NA	NA	ND	GenBank
Shandong	SDLZSheep01/2011	Sheep	2011.6	JQ693002	E	NA	NA	NA	NA	ND	GenBank
Shandong	SDLZDog01/2011	Dog	2011.6	JQ693003	D	NA	NA	NA	NA	ND	GenBank
Shandong	SDLZP01/2011	Homo sapiens	2011.6	JQ693004	D	NA	NA	NA	NA	ND	GenBank
Shandong	SDLZP02/2011	Homo sapiens	2011.6	JQ693005	F	NA	NA	NA	NA	ND	GenBank
Shandong	SDLZP05/2011	Homo sapiens	2011.6	JQ693008	F	NA	NA	NA	NA	ND	GenBank
Shandong	SDLZP07/2011	Homo sapiens	2011.6	JQ693010	F	NA	NA	NA	NA	ND	GenBank
Shandong	SDPLP01/2011	Homo sapiens	2011.6	JQ693013	D	NA	NA	NA	NA	ND	GenBank
Shandong	SDLZP03/2011	Homo sapiens	2011.6	JQ693006	F	NA	NA	NA	NA	ND	GenBank
Shandong	SDLZP04/2011	Homo sapiens	2011.6	JQ693007	F	NA	NA	NA	NA	ND	GenBank
Shandong	SDLZP06/2011	Homo sapiens	2011.6	JQ693009	F	NA	NA	NA	NA	ND	GenBank
Shandong	SDLZP08/2011	Homo sapiens	2011.7	JQ693011	F	NA	NA	NA	NA	ND	GenBank
Shandong	SDLZP09/2011	Homo sapiens	2011.7	JQ693012	F	NA	NA	NA	NA	ND	GenBank

Note: *, the SFTSV reassortants. NA, not available; ND, not determined.

Supplementary Table S3. Mean P distances between/within different SFTSV genotypes.

Segment	Clade	A	B	C	D	E	F
L	A	0.014±0.001					
	B	0.038±0.002	0.023±0.001				
	C	0.041±0.002	0.043±0.002	-			
	D	0.039±0.001	0.042±0.002	0.045±0.002	0.017±0.001		
	E	0.039±0.003	0.040±0.002	0.041±0.003	0.039±0.002	0.001	
	F	0.035±0.002	0.039±0.002	0.041±0.002	0.037±0.002	0.035±0.002	0.011±0.001
M	A	0.013±0.001					
	B	0.058±0.003	0.024±0.001				
	C	0.038±0.003	0.054±0.003	-			
	D	0.042±0.003	0.062±0.003	0.044±0.003	0.015±0.001		
	E	0.040±0.003	0.058±0.003	0.041±0.003	0.041±0.003	0.003±0.001	
	F	0.043±0.003	0.057±0.004	0.044±0.004	0.044±0.003	0.036±0.003	0.012±0.001
S	A	0.012±0.001					
	B	0.050±0.004	0.026±0.002				
	D	0.045±0.004	0.048±0.004		0.018±0.002		
	E	0.043±0.004	0.044±0.004		0.035±0.003	0.013±0.002	
	F	0.049±0.005	0.051±0.005		0.050±0.005	0.035±0.004	0.010±0.001

Supplementary Table S4. Parameter comparison between strict and relaxed molecular clocks in the evolutionary analyses of the three SFTSV segments.

	L strict	L relaxed	M strict	M relaxed	S strict	S relaxed
**Likelihood**	-28982.36	-28874.16	-16813.73	-16698.90	-9204.82	-9039.71
**Posterior**	-29971.15	-29936.11	-17809.12	-17608.37	-10350.66	-10220.63
**Prior**	-988.8	-998.15	-995.39	-996.79	-1145.84	-1100.08
**Population Size Scaled**	522.44	450.43	489.08	429.63	228.57	172.52

Supplementary Methods:

RT-PCR amplification and sequencing

Blood samples (200 μL) of patients were extracted for total nucleic acids (DNA and RNA) using the QIAamp MinElute Virus Spin Kit (Qiagen Sciences, USA) according to manufacturer's instructions. cDNA synthesis using total nucleic acids was performed using the PrimeScript RT-PCR Kit (Takara Bio Inc, Japan), according to manufacturer's instructions. Briefly, 8 μL total nucleic acids was mixed with 1 μL 10 mM dNTP Mixture and 1 μL 20 μM Random 6 primers, incubated at 65°C for 5 min, and then cooled down to 4°C. RT-PCR for cDNA synthesis was conducted by adding 4 μL 5×PrimeScript Buffer, 0.5 μL 40 U μL^{-1} RNase inhibitor, 5 μL RNase Free dH_2O, and 0.5 μL PrimeScript RTase, and incubated at 42°C for 60 min and then 70°C for 15 min.

Novel bunyavirus was detected using overlapping PCR in a 25 μL mixture containing 4 μL cDNA, 0.2 mM dNTP mixture, 1.5 mM $MgSO_4$, 0.5 U Platinum® Taq DNA Polymerase (Invitrogen, Life Technologies Corporation), and 0.2 μM primers [1,2]. Thermal cycling program was 94°C for 2 min, 35 cycles of 94°C for 30 s,

55°C for 30 s, and 68°C for 1 min, followed by 68°C for 5 min. Amplified products were detected by agarose gel electrophoresis and sequenced using an ABI Prism 3100 genetic analyzer (Applied Biosystems, Foster City, CA, USA). Whole genome sequences were assembled using the SeqMan package in the DNASTAR 6.0 software. SFTSV genomic sequences from 19 patients, including 17 from Zhoushan, one from Henan, and one from Anhui, were obtained for this study.

References:

1 Zhang, Y. Z. *et al.* Hemorrhagic fever caused by a novel tick-borne Bunyavirus in Huaiyangshan, China. *Zhonghua Liu Xing Bing Xue Za Zhi*. **32**, 209-220 (2011).

2 Li, S. *et al.* Sporadic case infected by severe fever with thrombocytopenia syndrome bunyavirus in a non-epidemic region of China. *Biosci Trends*. **5**, 273-276 (2011).
